# Clinically Relevant *KCNQ1* Variants Causing KCNQ1-KCNE2 Gain-of-Function Affect the Ca^2+^ Sensitivity of the Channel

**DOI:** 10.3390/ijms23179690

**Published:** 2022-08-26

**Authors:** Christiane K. Bauer, Tess Holling, Denise Horn, Mário Nôro Laço, Ebtesam Abdalla, Omneya Magdy Omar, Malik Alawi, Kerstin Kutsche

**Affiliations:** 1 Department of Cellular and Integrative Physiology, University Medical Center Hamburg-Eppendorf, 20246 Hamburg, Germany; 2 Institute of Human Genetics, University Medical Center Hamburg-Eppendorf, 20246 Hamburg, Germany; 3 Department of Medical Genetics and Human Genetics, Charité-Universitätsmedizin Berlin, Corporate Member of Freie Universität Berlin, Humboldt Universität zu Berlin and Berlin Institute of Health, 13353 Berlin, Germany; 4 Medical Genetics Unit, Hospital Pediátrico, Centro Hospitalar e Universitário de Coimbra, 3004-561 Coimbra, Portugal; 5 Department of Human Genetics, Medical Research Institute, Alexandria University, Alexandria 5422031, Egypt; 6 Genetics Department, Armed Forces College of Medicine (AFCM), Cairo 4460015, Egypt; 7 Department of Pediatrics, Faculty of Medicine, Alexandria University, Alexandria 5422031, Egypt; 8 Bioinformatics Core, University Medical Center Hamburg-Eppendorf, 20246 Hamburg, Germany

**Keywords:** Kv7.1, MiRP1, exome, K^+^ channelopathies, potassium channel, calmodulin, channel gating

## Abstract

Dominant *KCNQ1* variants are well-known for underlying cardiac arrhythmia syndromes. The two heterozygous *KCNQ1* missense variants, R116L and P369L, cause an allelic disorder characterized by pituitary hormone deficiency and maternally inherited gingival fibromatosis. Increased K^+^ conductance upon co-expression of KCNQ1 mutant channels with the beta subunit KCNE2 is suggested to underlie the phenotype; however, the reason for KCNQ1-KCNE2 (Q1E2) channel gain-of-function is unknown. We aimed to discover the genetic defect in a single individual and three family members with gingival overgrowth and identified the *KCNQ1* variants P369L and V185M, respectively. Patch-clamp experiments demonstrated increased constitutive K^+^ conductance of V185M-Q1E2 channels, confirming the pathogenicity of the novel variant. To gain insight into the pathomechanism, we examined all three disease-causing KCNQ1 mutants. Manipulation of the intracellular Ca^2+^ concentration prior to and during whole-cell recordings identified an impaired Ca^2+^ sensitivity of the mutant KCNQ1 channels. With low Ca^2+^, wild-type KCNQ1 currents were efficiently reduced and exhibited a pre-pulse-dependent cross-over of current traces and a high-voltage-activated component. These features were absent in mutant KCNQ1 channels and in wild-type channels co-expressed with calmodulin and exposed to high intracellular Ca^2+^. Moreover, co-expression of calmodulin with wild-type Q1E2 channels and loading the cells with high Ca^2+^ drastically increased Q1E2 current amplitudes, suggesting that KCNE2 normally limits the resting Q1E2 conductance by an increased demand for calcified calmodulin to achieve effective channel opening. Our data link impaired Ca^2+^ sensitivity of the KCNQ1 mutants R116L, V185M and P369L to Q1E2 gain-of-function that is associated with a particular KCNQ1 channelopathy.

## 1. Introduction

KCNQ1 is the founding member of the KCNQ or Kv7 family of voltage-gated K^+^ channels [1,2]. KCNQ1 channels activate reasonably fast and pass sustained outward current during long depolarizing pulses. Nevertheless, a concomitant hidden inactivation was inferred from the characteristic hook-like appearance of the tail currents recorded upon repolarization [3].

KCNQ1 channels associate with regulatory intracellular molecules, including phosphatidylinositol-4,5-bisphosphate (PIP2), adenosine triphosphate (ATP) and calmodulin (CaM), which are essential for KCNQ1 current generation [4,5,6,7]. CaM is constitutively bound to KCNQ1 and mandatory for functional channel assembly. Furthermore, it serves as a Ca^2+^ sensor and mediates acute stimulating effects of intracellular Ca^2+^ on KCNQ1 channels [4,5]. PIP2 is required to couple voltage sensor movement to pore opening [8] and can interact with CaM [9]. A hotspot of competitive binding is the proximal KCNQ1 C-terminus with its A and B helices, which are essential CaM binding sites in KCNQ channels [10,11]. Basic residues in the helix B of KCNQ1 have been shown to build a critical site where CaM is able to compete with PIP2 in a Ca^2+^-dependent manner in order to stabilize the KCNQ1 channel’s open state [9,12]. The authors assume a physiological role for this interaction in conditions with increased cytosolic Ca^2+^ and reduced PIP2, typically occurring after Gq-coupled receptor activation. Recently, the KCNQ1 S2–S3 linker has been identified as second channel site that interacts with either PIP2 or CaM [13,14,15]. Thus far, a Ca^2+^ dependence of this alternative interaction has not been described.

KCNQ1 is an extraordinary versatile channel, since ancillary subunits of the KCNE family induce drastic changes in the biophysical properties and amplitudes of the recorded KCNQ1 currents (reviewed, e.g., in [2,16,17,18,19]). The KCNQ1-KCNE1 (Q1E1) channel combination underlying the cardiac delayed rectifier current IKs, is by far the most extensively studied KCNQ1 channel. An enormous number of clinically relevant *KCNQ1* variants have been described in the context of cardiac arrhythmia, most of them leading to the long QT syndrome 1 by a loss of function reducing IKs [2,17]. Recently, two specific autosomal dominant *KCNQ1* missense variants, R116L and P369L, have been shown to underlie an allelic disorder characterized by growth hormone deficiency and maternally inherited gingival fibromatosis. Both variants result in significantly increased K^+^ conductance upon the co-expression of KCNQ1 mutant channels with KCNE2. This gain-of-function of the heteromeric KCNQ1-KCNE2 (Q1E2) channels has been suggested to underlie the clinical manifestations in the affected individuals [20]. Besides converting the KCNQ1 channel into a K^+^ leak channel with a preference for external acidification, KCNE2 exerts a strong inhibiting effect since macroscopic Q1E2 currents at depolarized potentials are significantly reduced compared to current amplitudes of KCNQ1 homomers [21,22,23,24]. Thus, by strongly increasing the constitutive K^+^ conductance, the *KCNQ1* variants R116L and P369L likely impair excitation-coupled hormone secretion and cause pituitary hormone deficiency in the affected individuals [20,25]. KCNE2 and KCNQ1 are expressed in hypothalamic and pituitary cells, raising the possibility that the two previously reported *KCNQ1* pathogenic variants may impact hormone secretion at different levels [20]. With co-expressed KCNE1, P369L-Q1E1 (but not R116L-Q1E1) channels yielded a moderately increased current density, which might explain why three out of four cardiologically examined individuals carrying the *KCNQ1* P369L variant exhibited a QTc time of less than the 2nd percentile [20].

Gingival overgrowth is not only found in individuals with the *KCNQ1* variants R116L and P369L, but also in individuals with another subgroup of channelopathies caused by K^+^ channel gain-of-function. Pathogenic variants in the disease genes *KCNH1*, *KCNK4* and *KCNN3* all establish a significant K^+^ conductance of the respective mutant channel in the more negative voltage range ([26,27,28,29,30], reviewed in [31]). Expression of KCNQ1 has recently been reported in human gingival fibroblasts and KCNQ1 was found to be upregulated in gingival tissue from individuals with non-syndromic hereditary gingival fibromatosis [32]. Thus, the *KCNQ1* variants R116L and P369L may have a proliferative effect on gingival fibroblasts and/or cranial neural crest cells, as proposed previously [20].

In this study, we aimed to identify the genetic cause underlying gingival overgrowth in an individual from one family and three affected members of the second family. By whole-exome sequencing, we detected the previously reported heterozygous *KCNQ1* variant p.P369L in the single individual, who also had short stature, and the novel heterozygous *KCNQ1* variant p.V185M in the three family members. To confirm the pathogenicity of the novel p.V185M variant, we investigated the functional impact of this S2–S3 linker variant on KCNQ1 homomers and on heteromeric channels formed by the association of KCNQ1 with KCNE1-3. In combination with KCNE2, V185M induced significantly increased current amplitudes, strongly supporting its pathogenicity. To gain further insight into the pathomechanism, we performed additional experiments on V185M and the previously reported disease-associated KCNQ1 mutants R116L and P369L, which revealed a resistance of all three mutant KCNQ1 channels to inhibition by low intracellular Ca^2+^. Altogether, our electrophysiological studies identified new aspects of the Ca^2+^ dependence of KCNQ1 wild-type (WT) channels and strongly suggest that the impaired Ca^2+^ sensitivity of the KCNQ1 mutant channels R116L, V185M and P369L is causally related to their gain-of-function when forming heteromers with KCNE2.

## 2. Results

### 2.1. Identification of a Previously Reported and Novel KCNQ1 Pathogenic Variant in Individuals with Gingival Overgrowth

We aimed to identify the genetic cause in individual 1 and three members of another family, a mother (individual 2), her son (individual 3) and sister (individual 4), who all had coarse facial features and early-onset gingival overgrowth. Individual 1 in addition had growth hormone deficiency and postnatal growth retardation. Trio whole-exome sequencing in individual 1, her healthy mother and healthy brother revealed the heterozygous *KCNQ1* (NM_000218.3) missense variant c.1106C>T/p.P369L in individual 1, which was absent in the mother and brother (Table A1 in Appendix A). This pathogenic variant has been reported in individuals with growth hormone deficiency and gingival fibromatosis [20]. Whole-exome sequencing followed by Sanger sequencing revealed the novel heterozygous *KCNQ1* variant c.553G>A/p.V185M in affected individuals 2 to 4 of the other family (Table A1 in Appendix A). The p.V185M variant is predicted to be damaging by the in silico tools detailed in Materials and Methods and likely underlies the phenotype in the three affected individuals.

### 2.2. Analysis of the Novel KCNQ1 Variant V185M Reveals Gain-of-Function

**Figure 1:**
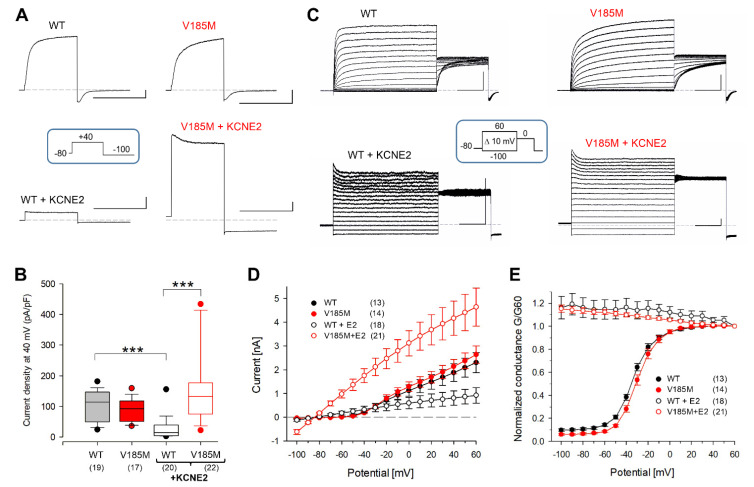
KCNQ1 variant V185M leads to high current densities in cells expressing KCNQ1-KCNE2 (Q1E2) heteromers. Whole-cell membrane currents were recorded in CHO cells expressing wild-type (WT) or V185M KCNQ1 channels alone or together with KCNE2. (**A**) Typical current traces recorded with a 500 ms test pulse to 40 mV. (**B**) Distribution of current densities determined with test pulses shortly after break-through. Asterisks indicate significant differences between groups; *** *p* < 0.001. (**C**) Typical families of current traces obtained with the indicated standard double pulse protocol, consisting of a 2 s variable P1 pulse and a constant P2 pulse to 0 mV. (**D**) Current amplitudes (means ± SEM) at the end of the 2 s P1 pulse plotted against P1 potential. (**E**) Conductance–voltage (GV) relation: normalized instantaneous P2 current amplitudes (means ± SEM) plotted against P1 potential. Numbers of experiments in (**B**,**D**,**E**) are given in parentheses; scale bars in (**A**,**C**) denote 0.5 nA and 0.5 s; standard extracellular (ECS) and intracellular solution (ICS). WT: WT KCNQ1; V185M: V185M-KCNQ1.

In a first set of experiments, we investigated the functional impact of the newly identified *KCNQ1* variant, V185M, on homomeric KCNQ1 channels and heteromeric Q1E2 channels to assess the pathogenicity of the variant (Figure 1). WT and mutant KCNQ1 channels were heterologously expressed in CHO cells, and membrane currents were recorded in the conventional whole-cell configuration. WT and mutant homomeric KCNQ1 channels exhibited voltage-dependent activation and considerably varying current amplitudes. WT and V185M channels did not differ with respect to median current density (Figure 1A,B), but they showed a slight difference in the voltage dependence of activation (Figure 1E; V0.5 and *k* values: −33.2 ± 1.1 mV and 9.2 ± 0.6 mV for WT, *n* = 13; −29.0 ± 1.6 mV and 8.6 ± 0.4 mV for V185M, *n* = 14; *p* = 0.0446 for V0.5 values). Since these data describe isochronal (2 s) activation, the shift towards more positive values is at least partially due to a slower activation time course of V185M (see also Figure S1).

Co-expression of the V185M mutant with the beta subunit KCNE2 led to significantly increased current densities compared to WT Q1E2 (Figure 1B,D). Despite the difference in amplitude, WT and V185M-Q1E2 currents were both characterized by the typical K^+^ leak conductance, with a slight time-dependent attenuation at more positive potentials (Figure 1C–E, [21]). Similarly, the co-expression with KCNE1 or KCNE3 resulted in typical current profiles of the respective heteromeric KCNQ1 channels [33,34,35,36], but in these experiments, the V185M variant had no effects on the voltage dependence of channel activation or current density (Figure S2). Thus, the most striking effect of the V185M variant was the gain-of-function of heteromeric Q1E2 channels, which fits well with the main effects of the pathogenic *KCNQ1* variants R116L and P369L described previously [20].

### 2.3. Mutant KCNQ1 Channels Exhibit Reduced Current Run-Down

In KCNQ1 channels, the coupling of voltage sensor activation to channel pore opening seems to crucially depend on the presence of several intracellular components, including PIP2 and ATP [6,7,37]. Consequently, the wash-out of cytosolic substances during whole-cell and especially during excised-patch recordings induces noticeable KCNQ1 current run-down [6,7]. We found that homomeric KCNQ1-V185M channels exhibited significantly less current run-down during the first five minutes of whole-cell recording compared to WT KCNQ1 channels when using the standard intracellular solution (ICS), containing a high level of ATP (5 mM) and low Ca^2+^ concentration (about 20 nM free Ca^2+^), but neither PIP2 nor CaM.

**Figure 2:**
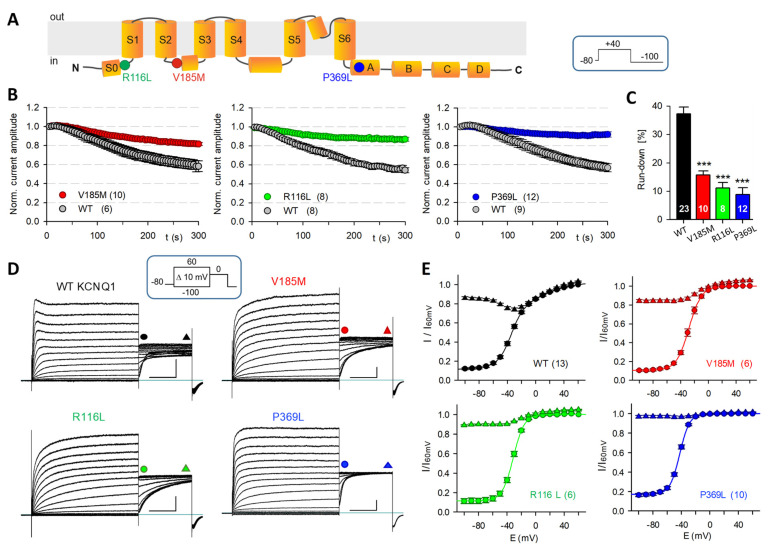
All three disease-associated *KCNQ1* variants lead to reduced early current run-down. (**A**) Scheme of the membrane topology of a KCNQ1 subunit with the location of the three studied amino acid substitutions indicated as colored dots. Transmembrane segments S1–S4 constitute the voltage-sensing domain, and S5 and S6 the pore domain. The cytoplasmic C-terminus harbors the proximal helices A and B and the distal helices C and D. (**B**) Whole-cell membrane currents were recorded in CHO cells expressing WT or mutant KCNQ1 channels using standard ECS and ICS. The time course of -current run-down was traced with a 500 ms test pulse to 40 mV every 5 s. Normalized current amplitudes (means ± SEM) for V185M, R116L and P369L are plotted together with the WT data of closely paralleled experiments. (**C**) Mean relative current amplitudes 3.5 to 4 min after the first measurement in the whole-cell configuration. Asterisks indicate significant differences to the combined WT data; *** *p* < 0.001. (**D**) Typical families of current traces, recorded after a 5 min tracing period with the indicated standard double pulse current–voltage (IV) protocol. Scale bars denote 250 pA and 0.5 s. (**E**) Mean (± SEM) normalized instantaneous P2 current amplitudes (GV relation) and current amplitudes measured at the end of the 1 s P2 pulse to 0 mV are plotted against P1 potential (see symbols in **D**). GV data points were fitted with a single Boltzmann function for the mutants and with a double Boltzmann function for WT KCNQ1 channels. Numbers of experiments are shown in parentheses.

Importantly, KCNQ1 channels with the previously described variants R116L and P369L that are located in the proximal N- or C-terminus, respectively, shared the reduced sensitivity of the new S2–S3 linker mutant V185M to early current run-down (Figure 2A–C). Typical families of whole-cell currents recorded after the 5 min period of run-down tracing are shown in Figure 2D. Analysis of the current amplitudes at the start and at the end of the constant P2 pulse to 0 mV revealed two remarkable differences between WT and mutant KCNQ1 channel activation: Firstly, the WT conductance–voltage (GV) data (P2 instantaneous current amplitudes) lacked saturation of current amplitudes at more positive potentials and had to be fitted with a double Boltzmann function, whereas single Boltzmann functions adequately described the voltage-dependent activation of the three mutant KCNQ1 channels (Figure 2E and Figure S3). Secondly, with incrementally depolarizing P1 pulses, only the WT current traces exhibited a striking cross-over during the constant P2 pulse, resulting in a minimum in the late P2 current amplitudes after P1 depolarization to −30 mV (Figure 2D,E). This slowing of WT KCNQ1 macroscopic current activation following partially activating voltage steps was not visible with P2 pulse potentials to −40 mV (Figure S3). The more negative P2 potential yielded only slight differences in the parameter describing voltage-dependent isochronal channel activation (Figure S3), faithfully reproducing the two components of WT channel activation. With a slope factor of about 25 mV, the additional high-voltage-activated (HVA) WT KCNQ1 current component (V0.5 values near 0 mV) exhibited a low voltage sensitivity.

### 2.4. High Intracellular Ca^2+^ and CaM Counteract Early Current Run-Down

**Figure 3:**
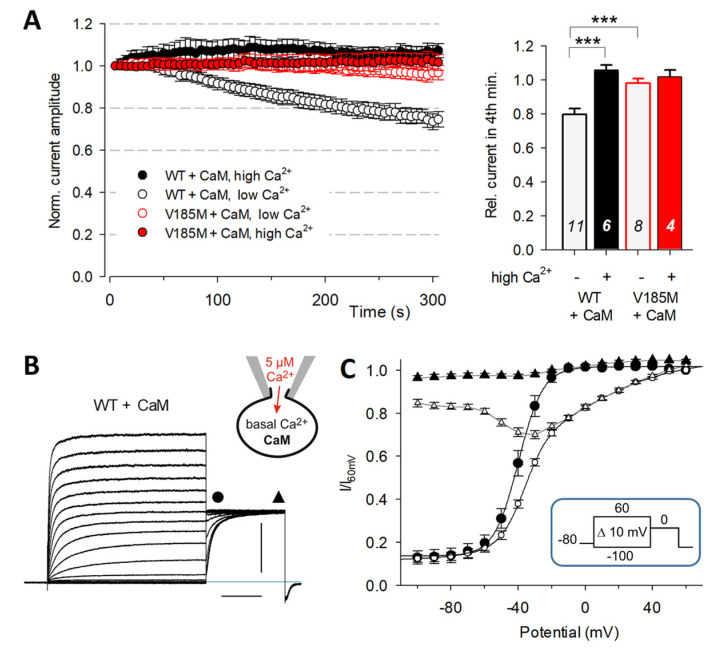
In cells co-expressing calmodulin (CaM), high intracellular Ca^2+^ abolishes early current run-down and characteristic WT KCNQ1 properties. Whole-cell recordings were performed with different Ca^2+^ concentrations of ICS in CHO cells expressing the KCNQ1 channels, together with CaM. (**A**) Time course of current amplitudes traced with a 500 ms test pulse to 40 mV. Mean normalized current amplitudes for WT and V185M-KCNQ1 channels from experiments with standard low-Ca^2+^ ICS and high-Ca^2+^ (5 µM) ICS. Numbers of experiments are given in the bar plot, which shows mean (+SEM) relative current amplitudes after 3.5 to 4 min. Asterisks indicate significant differences; *** *p* < 0.001. (**B**) Example of WT KCNQ1 current traces, recorded in a cell co-expressing CaM > 5 min in whole-cell configuration using 5 µM-Ca^2+^ ICS. Scale bars denote 1 nA and 500 ms. (**C**) Mean (± SEM; *n* = 7) normalized instantaneous P2 current amplitudes (GV relation, filled circles) and current amplitudes measured at the end of the 1 s P2 pulse to 0 mV (filled triangles) are plotted against the P1 potential. Filled symbols: data for WT KCNQ1 channels co-expressed with CaM and recorded with high-Ca^2+^ ICS, as shown in (**B**). Open symbols: data of seven closely paralleled control experiments on WT KCNQ1 without CaM co-expression using standard low-Ca^2+^ ICS.

To explore the reason behind the strong current run-down of WT KCNQ1 channels, we used modified intracellular solutions and investigated the effect of CaM co-expression. Adding PIP2 (100 µM PIP2DiC8) to the standard ICS was unable to impede the WT KCNQ1 current run-down in five of the six experiments (Figure S4), suggesting only a minor role of PIP2 concentration changes during the first minutes of whole-cell configuration. In contrast, raising the ICS Ca^2+^ concentration to 5 µM prevented the current run-down in cells co-expressing WT or V185M-KCNQ1 channels, together with CaM (Figure 3A). Notably, with co-expressed CaM and high intracellular Ca^2+^, WT KCNQ1 GV curves obtained after more than 5 min in the whole-cell configuration were well fitted with a single Boltzmann function (V0.5 = −39.6 ± 2.0 mV; *k* = 6.6 ± 0.2 mV; *n* = 7) and P2 current traces did not show cross-over (Figure 3B,C).

**Figure 4:**
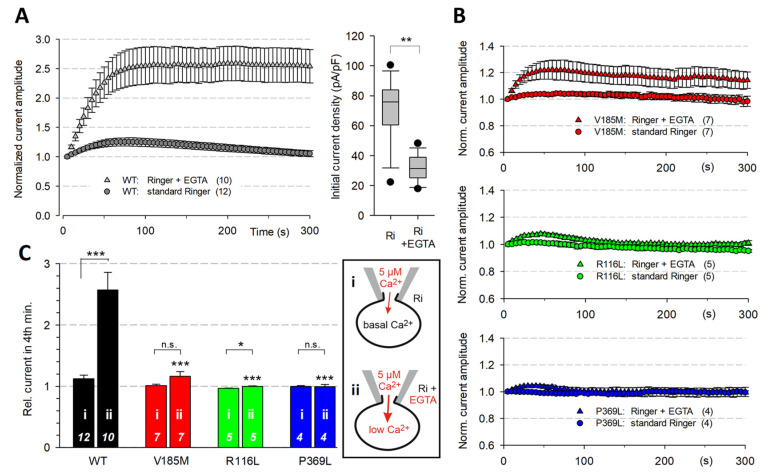
Ca^2+^ sensitivity of WT KCNQ1 channels is blunted in the mutant channels. Whole-cell recordings were performed using high-Ca^2+^ ICS in CHO cells expressing KCNQ1 channels without overexpression of CaM. Cells were pre-incubated for at least 30 min in normal (i) or EGTA-Ringer solution (ii). (**A**) Time course of WT KCNQ1 current amplitudes traced with a 500 ms test pulse to 40 mV (left panel). Normalized current amplitudes (means ± SEM) for experiments in standard or EGTA-Ringer as ECS. Initial current densities were significantly lower in EGTA-Ringer compared to standard ECS (right panel; ** *p* < 0.01). (**B**) Time course of normalized current amplitudes (means ± SEM) for experiments on V185M-, R116L- and P369L-KCNQ1 channels in standard or EGTA-Ringer as ECS. (**C**) Comparison of the effects of pre-incubation in EGTA-Ringer on the changes in relative current amplitude for WT and mutant KCNQ1 channels. Asterisks directly above a box indicate significant differences in relative current amplitudes of mutant KCNQ1 channels compared to the WT data obtained under the same experimental conditions. Other asterisks indicate significant differences between conditions “i” and “ii” (see schematic drawings). *** *p* < 0.001; * *p* < 0.05; n.s., not significant; numbers of experiments are shown in the bars.

When using the standard low-Ca^2+^ ICS, overexpression of CaM was unable to completely prevent current run-down in WT KCNQ1 channels (Figure 3A; run-down of 20.4 ± 3.3% compared to 37.3 ± 2.3% without CaM co-expression; *p* = 0.0003). In contrast, the high-Ca^2+^ ICS stabilized WT KCNQ1 current amplitudes for several minutes, in CHO cells with endogenous CaM levels, too, indicated by a mean relative 4 min current amplitude of 112 ± 6% (Figure 4A,C*)*. In all these experiments, changes in the intracellular Ca^2+^ concentration via the patch pipette were inevitably accompanied by a more or less delayed concomitant washout of PIP2 and other factors. To better isolate a possible effect of Ca^2+^ on the availability of WT and mutant KCNQ1 channels, we used two different strategies to lower the intracellular Ca^2+^ level in intact CHO cells.

### 2.5. Mutant KCNQ1 Channels Exhibit Impaired Ca^2+^ Sensitivity

Pre-incubation in a low-Ca^2+^ bath solution (EGTA-Ringer) prior to whole-cell recording for at least 30 min resulted in significantly decreased initial WT KCNQ1 current amplitudes and a time-dependent current increase to more than twofold the initial value upon equilibrating the cells with 5 µM-Ca^2+^ ICS (Figure 4A). This pre-incubation effect was much less pronounced in cells expressing KCNQ1-V185M and almost absent in cells expressing the KCNQ1 channels with R116L or P369L (Figure 4B,C).

**Figure 5:**
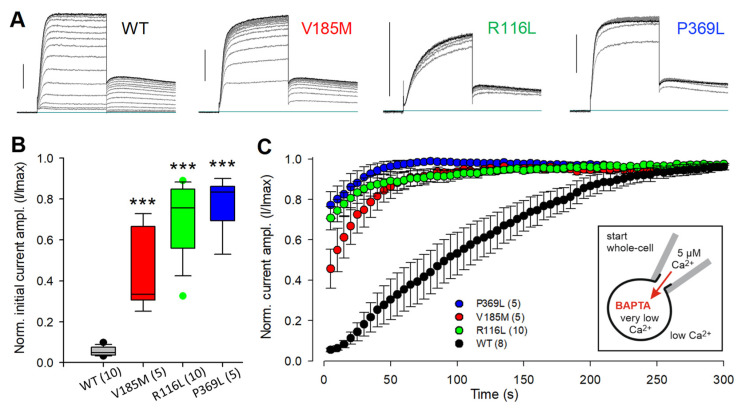
BAPTA experiments confirm the different sensitivities of WT and mutant KCNQ1 channels to the inhibiting effect of low intracellular Ca^2+^. Two hours prior to the start of the recording, cells were incubated for 1 h in medium containing 10 µM BAPTA-AM followed by a 1-h wash period in normal CHO culture medium in the incubator at 37 °C. Whole-cell recordings were performed in EGTA-Ringer with high-Ca^2+^ ICS in CHO cells expressing WT or mutant KCNQ1 channels. (**A**) Overlays of the first and then every fourth current trace recorded with a 500 ms test pulse to 40 mV, followed by a 1 s pulse to −40 mV; test pulse interval 5 s. To illustrate the current run-up at 5 min after establishing the whole-cell configuration, the respective last trace is shown in black. Vertical bars denote 0.5 nA. (**B**) Boxplot of initially recorded current amplitudes, normalized to the respective maximal current amplitude during a 5 min recording period. Asterisks indicate significant differences to WT data; *** *p* < 0.001. Numbers of experiments are given in parentheses. (**C**) Time course of current run-up in BAPTA-containing cells during Ca^2+^ loading via the recording pipette. Data are shown as means ± SEM of normalized current amplitudes.

To reduce the intracellular Ca^2+^ levels in intact cells even further, CHO cells were loaded with BAPTA by BAPTA-AM pre-incubation. This treatment almost abolished the initial WT KCNQ1 current and led to drastic relative increases in K^+^ current amplitudes during Ca^2+^ loading via the recording pipette (Figure 5A–C). BAPTA loading of cells expressing the mutant KCNQ1 channels led to significantly higher relative initial current amplitudes and a much faster time course of current restoration upon Ca^2+^ loading. Again, the R116L and P369L mutants were less affected by the reduction of intracellular Ca^2+^ than the V185M mutant. In summary, these data strongly suggest that all three identified missense variants impair the Ca^2+^ sensitivity of the KCNQ1 channel and stress the necessity of at least normal resting Ca^2+^ levels for effectively coupling voltage sensor activation to channel opening in WT KCNQ1 channels.

### 2.6. Impaired Ca^2+^ Sensitivity of KCNQ1 Mutant Channels Causes Gain-of-Function of Heteromeric KCNQ1-KCNE2 Channels

**Figure 6:**
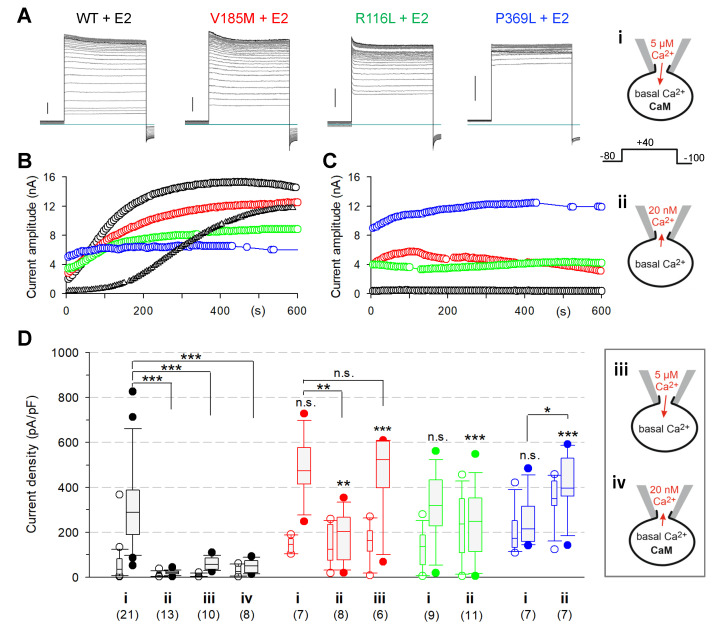
High intracellular Ca^2+^-CaM levels counteract the differences in current densities between WT and mutant Q1E2 channels. Whole-cell recordings performed in standard Ringer on cells co-expressing KCNE2 with WT or mutant KCNQ1. In two sets of experiments, CaM was additionally co-expressed (condition i and iv, see schematic drawings). The ICS contained either a high (i and iii) or a low (ii and iv) Ca^2+^ concentration. (**A**) Overlays of the first and then every fourth current trace recorded with 500 ms test pulses to 40 mV from cells co-expressing CaM and using high-Ca^2+^ ICS (“i”). The respective last trace within 10 min after establishing the whole-cell configuration is shown in black. Vertical bars denote 2 nA. (**B**) Time course of current amplitudes for the experiments shown in (**A**) (open circles). In addition, a second WT Q1E2 experiment is given (black triangles) to illustrate the variability in the time courses of the current increase. (**C**) Time course of current amplitudes from exemplary experiments performed with standard condition “ii”. (**D**) Boxplot of initial Q1E2 current densities (thin boxes, open circles for all outliers) and maximal Q1E2 current densities (broad boxes, filled circles for all outliers) obtained in differing experimental conditions (i–iv). Color code as shown in (**A**). Asterisks directly above a box indicate significant differences in maximal current density for the mutant Q1E2 compared to WT Q1E2 data obtained under the same experimental conditions. Other asterisks indicate significant differences in maximal current density between condition “i” and another recording condition. *** *p* < 0.001; ** *p* < 0.01; * *p* < 0.05; n.s., not significant. Numbers of experiments are given in parentheses.

To explore whether the altered Ca^2+^ sensitivity of the channel mutants might be functionally related to the observed gain-of-function of the respective Q1E2 channel complexes, we assessed the effects of CaM co-expression and intracellular Ca^2+^ loading on WT and mutant Q1E2 channels (Figure 6A,B,D; condition “i”). Loading CaM-co-expressing cells with Ca^2+^ via the patch pipette resulted in more or less delayed dramatic increases in the current amplitude for WT Q1E2 channels, eliminating the characteristic difference to the mutant Q1E2 channels. Neither high Ca^2+^ alone nor CaM overexpression alone were sufficient to induce similarly high WT Q1E2 current densities (Figure 6D; “iii” and “iv”). Moreover, Ca^2+^ loading of V185M-Q1E2 channel-expressing cells significantly boosted the recorded current amplitudes, but this effect did not require overexpression of CaM. Parallel control measurements with standard low-Ca^2+^ ICS and endogenous CaM levels (Figure 6C,D; condition ii) confirmed the finding ([20] and this study, Figure 1) that all three *KCNQ1* variants resulted in significantly increased Q1E2 current amplitudes compared to WT Q1E2. With overexpressed CaM, R116L-Q1E2 current amplitudes also clearly increased during high Ca^2+^ loading. In contrast, P369L-Q1E2 current tracing exhibited a moderate rise in amplitude, with or without elevated Ca^2+^ and CaM, suggesting that this change in amplitude was independent of the intracellular Ca^2+^ concentration. Moreover, initial and maximal current densities tended to be even smaller with co-expressed CaM and high Ca^2+^, suggesting a complete loss of the stimulating effect of Ca^2+^-CaM by the P369L variant.

### 2.7. Corroboration of the Crucial Role of Ca^2+^ in Q1E2 Channel Activation in Somato-Mammotroph Anterior Pituitary Cells

**Figure 7:**
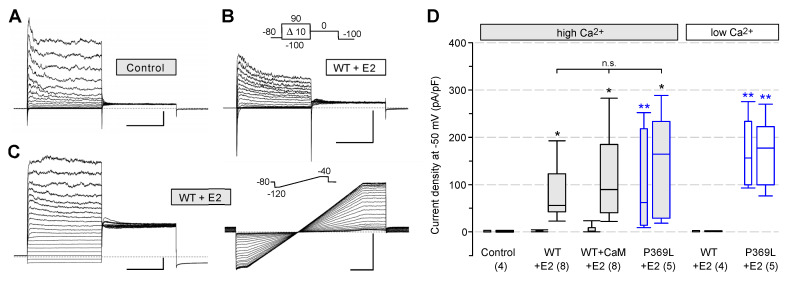
High intracellular Ca^2+^ activates WT Q1E2 channels expressed in GH_3_/B_6_ anterior pituitary cells. Whole-cell recordings were performed in native GH_3_/B_6_ somato-mammotroph cells (Control) or cells overexpressing KCNE2, together with either WT or P369L-KCNQ1. In one set of WT KCNQ1 experiments, CaM was additionally co-expressed. The ICS contained either a high (5 µM) or a low (20 nM) Ca^2+^ concentration. (**A**) Family of current traces recorded from a native GH_3_/B_6_ cell with the pulse protocol shown in (**B**) using high-Ca^2+^ ICS. (**B**) Membrane currents recorded with low-Ca^2+^ ICS in a cell overexpressing WT Q1E2. Peak Na^+^ inward currents are truncated. (**C**) Membrane currents recorded with high-Ca^2+^ ICS in a cell overexpressing WT Q1E2. Prior to the IV protocol (family of current traces, left panel), activation of WT Q1E2 channels was traced for 5 min by applying a voltage ramp ranging from −120 mV to −40 mV every 2 s. An overlay of the first and then every fifth current trace is shown (right panel). Scale bars in (**A**–**C**) denote 1 nA and 100 ms. (**D**) Boxplot of initial current densities at −50 mV (thin boxes) and maximal current densities after 4−5 min in the whole-cell configuration (broad boxes). Black asterisks directly above a box indicate significant differences to the control data obtained with high-Ca^2+^ ICS in native GH_3_/B_6_ cells. Blue asterisks indicate significant differences compared to the respective WT Q1E2 data recorded with the same ICS. ** *p* < 0.01; * *p* < 0.05; n.s., not significant. Numbers of experiments are given in parentheses.

Next, we examined the Q1E2 channel activation by highly calcified CaM in a rat pituitary tumor cell line. GH_3_/B_6_ cells are clonal growth hormone- and prolactin-producing cells and exhibit electrical activity-coupled hormone secretion [25]. Among a number of different K^+^ channel alpha and beta subunits, GH_3_/B_6_ cells endogenously express KCNQ1 and KCNE2, but not KCNE1 [38,39]. Membrane currents recorded from native GH_3_/B_6_ cells using high-Ca^2+^ ICS demonstrated a low total membrane conductance at more negative membrane potentials to −40 mV (Figure 7A,D), suggesting a limited number of endogenous Q1E2 and calcium-activated small-conductance K^+^ (SK) channels. Activation of calcium-activated big-conductance K^+^ (BK) channels by intracellular Ca^2+^ was apparent at increasingly positive potentials. Heterologous WT Q1E2 expression did not result in increased membrane conductance at negative potentials with low-Ca^2+^ ICS (Figure 7B,D), but loading the cells with high Ca^2+^ strongly increased the K^+^ conductance in the negative potential range, most probably carried by Q1E2 channels (Figure 7C,D). In contrast to our results for the CHO cells, the high-Ca^2+^-activated Q1E2 current density did not depend on additional CaM overexpression (Figure 7D). Expression of the KCNQ1 mutant P369L, together with KCNE2, yielded high initial as well as sustained current densities at −50 mV, even with a low intracellular Ca^2+^ concentration (Figure 7D), confirming this significant and most probably pathogenic difference to WT Q1E2 channels in GH pituitary cells.

Together, our results provide experimental evidence that KCNE2 association with WT KCNQ1 channels exerts a drastic sustained inhibition of the Q1E2 channel availability in resting CHO and GH pituitary cells, and that CaM requires high Ca^2+^ to effectively relieve this inhibition. In contrast, the three analyzed KCNQ1 mutants that were found to exhibit an impaired Ca^2+^ sensitivity as homomers, mediate a significantly increased resting K^+^ conductance when associated with KCNE2. In CHO cells, R116L- and V185M-Q1E2 mutant channels can still be stimulated by high Ca^2+^, while this is not the case for P369L-Q1E2. These data suggest that the three clinically relevant *KCNQ1* variants may differ in their gain-of-function effects on the Q1E2 channel properties.

## 3. Discussion

With the identification of the recurrent *KCNQ1* variant P369L and the newly identified V185M variant, we confirm that specific heterozygous *KCNQ1* missense variants (R116L, P369L and V185M) cause gingival overgrowth, with or without postnatal growth retardation (our data and [20]). Maternal inheritance of the *KCNQ1* variant was identified in individual 3 but could not be determined in individuals 1, 2 and 4.

Our analysis of WT KCNQ1 channels demonstrated an efficient inhibition of the K^+^ conductance by reduced intracellular Ca^2+^ levels. In CHO cells, CaM overexpression combined with high intracellular Ca^2+^ drastically increased WT Q1E2 currents, suggesting that KCNE2 normally limits the resting Q1E2 conductance by shifting the required threshold for channel opening towards higher concentrations of both Ca^2+^ and CaM. The KCNQ1-R116L, -P369L and -V185M mutant channels lack the characteristic Ca^2+^ sensitivity of WT KCNQ1 channels, suggesting that altered Ca^2+^ sensitivity of the KCNQ1 mutants underlies the prominent gain-of-function of heteromeric Q1E2 channels. The crucial role of Ca^2+^ in determining the macroscopic Q1E2 conductance was confirmed in somato-mammotroph anterior pituitary cells. Moreover, constitutively active P369L-Q1E2 channels induced a high resting K^+^ conductance in GH_3_/B_6_ cells, which is expected to inhibit electrical activity-coupled hormone secretion by shifting the resting potential towards the K^+^ equilibrium potential and thereby reducing the voltage-dependent Ca^2+^ influx [25]. Our data causally link impaired Ca^2+^ sensitivity of the KCNQ1 mutants to postnatal growth retardation and/or gingival overgrowth, characterizing a particular *KCNQ1* channelopathy.

Our study provides mechanistic insight into the disease-causing gain-of-function of mutant KCNQ1 channels when associated with KCNE2 and strengthens the data published by Tommiska et al. [20]. Thus, the selective pharmacological inhibition of Q1E2 mutant channels appears to be a promising therapeutic approach for treating this ultra-rare *KCNQ1*-related disease. To date, no officially approved drug with a potent Q1E2 channel-blocking activity is known [40]. The feasibility of finding a Q1E2-selective channel blocker is suggested by the action of the compound IKs124, a derivative of the KCNQ1 channel blocker chromanol 293B [22]. Moreover, extracts from Californian plants used in traditional botanical medicine have recently been shown to exert agonistic or antagonistic subunit-dependent effects on KCNQ channels [41]. We expect that the newly identified pronounced activation of WT Q1E2 channels by high intracellular Ca^2+^ can be effectively exploited to test potential channel blockers.

**Figure A1:**
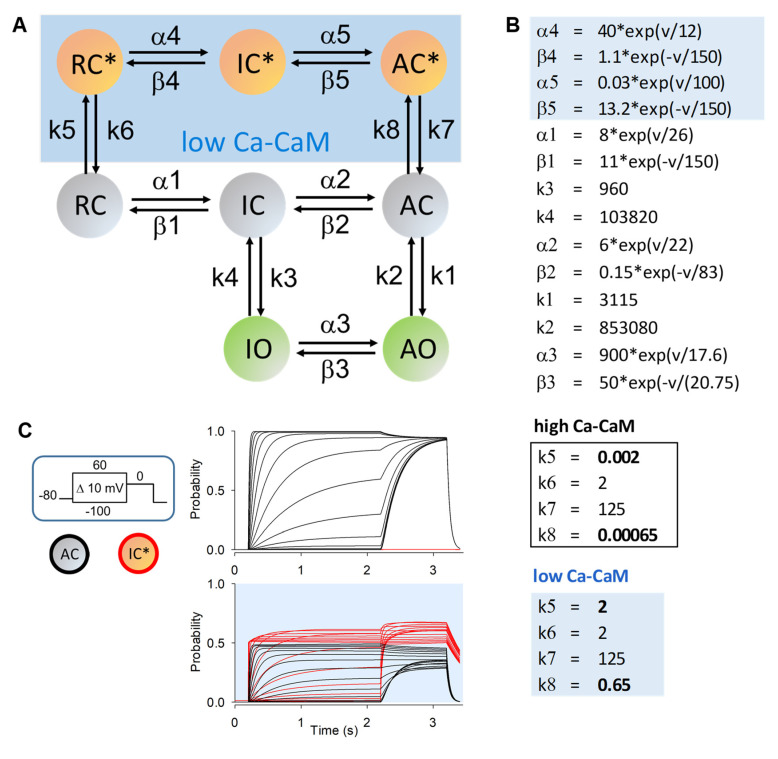
A Markov model considering the Ca^2+^ sensitivity of the KCNQ1 WT channel. (**A**) The five-state kinetic model in the lower part (white background) of the scheme applies to homomeric KCNQ1 channels with saturating concentrations of Ca^2+^ and CaM (high Ca-CaM). To account for the effects of reduced Ca^2+^, three closed states (*) were added without direct access to the open states IO and AO (low Ca-CaM; blue background). From IC*, channels have to pass through AC* to enable channel opening. (**B**) Model parameter used to describe WT KCNQ1 channel behavior with low and high intracellular Ca-CaM. k5 and k8 determine the access to *states; the ratio of k5/(k5+k6) mirrors the amount of current reduction induced by decreased levels of calcified CaM. (**C**) With low Ca-CaM, the potential- and time-dependent accumulation of channels in the AC state (black traces) is significantly reduced due to the high occupancy of the IC* state (red traces). “R”, “I” and “A” indicate the resting, intermediate or activated positions of the voltage sensor; “C” and “O” indicate the closed or open states of the channel. Simulated KCNQ1 current traces resulting from this model are shown in Figure S1 (deactivation protocol) and Figure S6 (Ca^2+^ dependence: activation protocol).

By demonstrating the stimulating effects of Ca^2+^ on homomeric WT KCNQ1 channels, including increased current amplitudes and a leftwards shift in the voltage dependence of channel activation, our results confirm previously reported data [4,5]. It has been suggested that CaM and Ca^2+^ relieve KCNQ1 channel inactivation [4]. We assume that KCNQ1 channels are subject to different types of “inactivation”: one type of inactivation generates the characteristic hook upon repolarization, which was initially described by a linear gating model with two open and a final inactivating state [3,42] and most recently by a non-linear simplified five-state model with two sequentially activated voltage sensor states (I: intermediate, A: fully activated), both allowing distinct channel opening [43]. Another type of channel inactivation is due to a shortage of accessory factors, as suggested for a lack of PIP2, resulting in a decoupling of voltage sensor activation from pore opening [8,14]. Concerning the role of Ca^2+^-CaM, our data add two properties to the characteristics of macroscopic KCNQ1 currents recorded with low intracellular Ca^2+^: the cross-over phenomenon, which is reminiscent of U-type inactivation [18,44], and the presence of an additional HVA current component, reminiscent of the biphasic movements of the KCNQ1 voltage sensor measured by voltage clamp fluorimetry (F_main_ and F_high_, [45]; reviewed in [18]). The detection of pre-pulse-dependent cross-over requires a special pulse protocol (Figure S3), and often, the HVA current component may be obscured by a concomitant current run-down (Figure S5). An unusual “cross-over of current traces” has previously been described for the *Xenopus* oocyte heteromeric I-SK channels formed by endogenous KCNQ1 channel subunits and heterologously expressed KCNE1 subunits [46]. The authors postulated the presence of different activation pathways that generate this cross-over, and this behavior was later simulated by adding a closed inactivated state branching off from a sequential gating scheme [47]. We used the concept of different activation pathways to reproduce the Ca^2+^ dependence of WT KCNQ1 channels in a gating model. We extended a modified version of the five-state model of Hou et al. [43] by adding three additional closed states (C*) occupied at insufficient Ca^2+^-CaM levels and explored whether a partial block of the IC–IO transition could mimic the observed effects of low Ca^2+^ on WT KCNQ1 channels. The extended model (Figure A1 in Appendix B) is characterized by time- and voltage-dependent accumulation in the intermediate “inactivated” IC* state, with a limited possibility of exit from the inactivated state to the fully activated state. Model simulations of WT KCNQ1 current traces reproduced the occurrences of the HVA current component and the pre-pulse-dependent cross-over of current traces at the P2 pulse to 0 mV (Figure S6). The model implied that the cross-over phenomenon develops over the duration of the P1 pulse, which was verified experimentally by using shorter P1 pulses (Figure S6). As an aside, it is worth noting that changing the transitions between AC* and AC in favor of AC* occupancy reproduces the inactivating current profile of LQT1 KCNQ1 mutants with a CaM-binding deficiency [5]. Based on our experimental data and the extended Markov model, we propose that Ca^2+^ is required to efficiently couple the intermediate voltage sensor state to channel opening and to facilitate the voltage-dependent transition towards the fully activated state.

It is assumed that the effects of Ca^2+^ on KCNQ1 currents are mediated by CaM, which is constitutively associated with the channel in a 4:4 stoichiometry [4,13]. CaM is able to bind simultaneously to the C-terminal domain (CTD) with helices A and B and to the S2–S3 linker as part of the voltage-sensing domain (VSD) [13]. Importantly, both sites can also interact with PIP2, which is needed for voltage-dependent channel opening [8,13,14,15]. Positive charges are crucial in forming PIP2 binding pockets [8], and two basic residues in helix B (K526 and K527) form a critical site of competitive CaM and PIP2 binding [9]. Moreover, this competition was found to be Ca^2+^-dependent. The authors assumed a resting condition, where helix B interacts with PIP2 and the calcified CaM N-lobe, and where the uncalcified CaM C-lobe concomitantly binds to helix A. With increased cytosolic Ca^2+^ and reduced PIP2, it was suggested that the now-calcified CaM C-lobe unbinds from helix A and the CaM N-lobe lobe replaces PIP2 at helix B to enable channel opening during PIP2 depletion [9]. Another structural model dealing with the Ca^2+^ sensitivities of different Kv7 channels involves a major CaM C-lobe switch as the critical step in Ca^2+^-dependent facilitation of KCNQ1 channel opening, as well as in Ca^2+^-dependent inhibition of KCNQ4 channels [48]. Of note, in contrast to KCNQ1, rises in Ca^2+^ inhibit homomeric KCNQ2, KCNQ4 and KCNQ5 channels and heteromeric KCNQ2-Q3 “M” channels [49].

**Figure A2:**
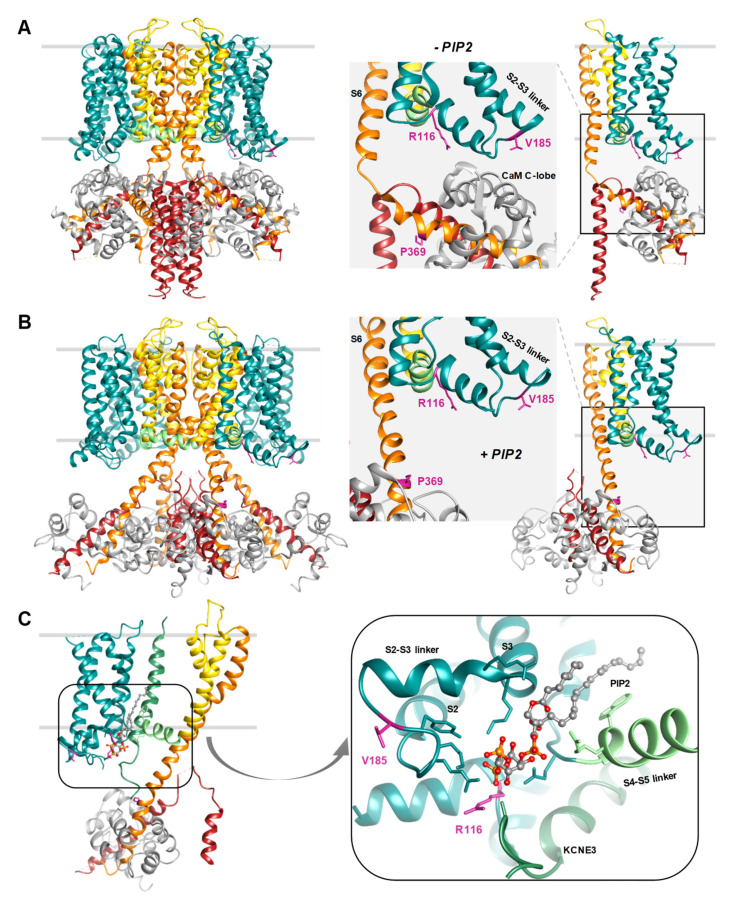
Structure models of the KCNQ1-CaM complex in the PIP2-free and the PIP2-bound state and location of amino acid residues R116, V185 and P369. (**A**,**B**) Visualization of KCNQ1 (transmembrane helices S1 to S4 in turquoise, S4–S5 linker in light green, S5 in yellow, S6 and helix A in orange and helices B and C in red) and CaM (in grey) in the tetrameric KCNQ1-KCNE3-CaM complex in the PIP2-free state (**A**, based on pdb 6V00) and in the PIP2-bound state (B), based on pdb 6V01) and orientation in the membrane (gray lines). KCNE3 has been removed for clarity. Amino acid residues R116, V185 and P369 are highlighted in magenta and shown as sticks in a single KCNQ1 subunit. On the left, KCNQ1 and CaM are shown as a tetrameric channel complex. On the right, one KCNQ1 subunit with the highlighted amino acid residues R116, V185, and P369 and one CaM subunit are shown. The structural rearrangement of S6 and helix A, which form two helices connected by a loop in the PIP2-free state (**A**, right panel) to a continuous straight helix in the PIP2-bound state, is shown (**B**, right panel). The insets in the middle show a magnification to highlight the location of R116, V185 and P369, as well as the close proximity of R116 and V185 to the CaM C-lobe in the PIP2-free state (**A**), but not in the PIP2-bound (**B**) state. (**C**) Structure model of one KCNQ1, CaM and KCNE3 (green) subunit of the tetrameric KCNQ1-KCNE3-CaM complex in the PIP2-bound state and orientation in the membrane (based on pdb 6V01). PIP2 is shown as balls and sticks. The inset on the right shows a magnified reoriented view of the PIP2 binding pocket. R116 and V185 are highlighted in magenta. Side chains of residues in the vicinity of PIP2 are shown as sticks.

The S2–S3 linker of KCNQ1 emerged as a second hotspot of alternative CaM and PIP2 binding [13,14,15]. The KCNQ1 S2–S3 linker is nine amino acids longer than in most other Kv channels [2]. This S2–S3 linker extension (G179–L187) forms a loop, which constitutes a CaM binding site and harbors basic amino acids (R181, K183), which contribute to a positively charged PIP2 binding pocket [13,14,15]. Cryo-EM structures of CaM-bound KCNQ1 obtained in the presence or absence of PIP2 [13,14] showed that, without PIP2, the S2–S3 linker interacts with the CaM C-lobe. In the presence of PIP2, the interaction between the S2–S3 linker and the CaM C-lobe is lost and is accompanied by a reorientation of CaM toward the central axis of the channel complex (Appendix B: Figure A2A,B). It has been speculated that PIP2 serves to displace CaM from the S2–S3 linker, so that the C-terminus can reorient itself for channel opening [18]. Molecular dynamics simulations combined with electrophysiology data suggest that, in the presence of PIP2, the CaM C-lobe transiently interacts with the S2–S3 linker during voltage-dependent channel gating [15]. The authors proposed a gating scheme, where the voltage sensor state controls the subsequent CaM and PIP2 binding to the S2–S3 linker. CaM binding is favored with the voltage sensor being in the resting or intermediate state, and the transition to the fully activated state is suggested to trigger the S2–S3 linker to switch from binding CaM to binding PIP2 in order to stabilize the AO state. We assume an important role of Ca^2+^ in the CaM “switch” described by Kang et al. [15]. Our present data highlight the S2–S3 linker as an important site, where CaM binding not only stabilizes the IC state but might also “lock” the channel in this conformation when there is an insufficient availability of Ca^2+^ (“inactivated” IC* state; Figure A1).

Altogether, the observed Ca^2+^ effects on KCNQ1 currents might arise from a highly complex interplay between CaM and PIP2 binding in Ca^2+^-dependent competition at two different sites of the channel subunits (helix B and S2–S3 linker) and interdependencies of the different voltage sensor states with the actual binding partner. Such a high complex regulation opens several possibilities with respect to the effects on the Ca^2+^ sensitivity of the KCNQ1 channel, which is reflected by the localization of the three clinically relevant *KCNQ1* missense variants: Arg116, located in the N-terminus, Val185, in the S2–S3 linker, and Pro369, in the C-terminus.

V185 is located in the loop region of the S2–S3 linker. This valine is not conserved within the Kv7 family; instead, basic amino acids (R or K) are found in KCNQ2–5 [50] that probably promote the PIP2 binding to the S2–S3 linker in these KCNQ channels [51] and take part in the CaM regulation of KCNQ4 gating [52]. Both V185 and P369 have been demonstrated to play a role in the CaM dependence of KCNQ1 channel opening characteristics [15]. If CaM binding to the S2–S3 linker stabilizes the resting and intermediate positions of the voltage sensor, amino acid changes which impair CaM binding to the S2–S3 linker should promote pore opening in the fully activated voltage sensor state, resulting in a more pronounced slowly activating current component at depolarized potentials [15]. Thus, the slower activation of KCNQ1-V185M channels observed in the present study (Figure S1) may also suggest a destabilization of the intermediate state, which favors the transition to the fully activated voltage sensor position.

P369 is located in the proximal C-terminus between pre-helix A and helix A, where it generates a slight kink [11]. Remarkably, the region beyond the transmembrane domain S6 becomes helical in the presence of PIP2, resulting in a single continuous helix combining S6 and helix A ([14]; Figure A2A,B). Most intriguingly, Kang et al. [15] found that P369 is required to transfer effects of altered S2–S3 linker CaM binding to channel opening. It is tempting to speculate that changes in Ca^2+^ levels, which influence CaM-PIP2 interactions at the S2–S3 linker, also need P369 to affect channel opening. Hence, the pronounced insensitivity to Ca^2+^ of P369L homomeric KCNQ1 and heteromeric Q1E2 channels could rely on a downstream mechanism circumventing the primary Ca^2+^-sensing process. The functional importance of a proline at this position for all KCNQ family members is indicated by the gain-of-function of the *KCNQ5* variants P369R and P369T, affecting the proline analogous to KCNQ1 P369 [53,54]. Heterozygous gain- and loss-of-function variants in *KCNQ5* cause pediatric neurological disorders of different severities [53,54,55].

R116 is located in a juxtamembranous region of the proximal N-terminus harboring amino acids, which critically affect channel trafficking [56]. R116 is considered to be part of a positively charged PIP2 binding pocket also comprising basic amino acids of the S2–S3 linker and the S4–S5 linker of KCNQ1 (Figure A2C; [14], reviewed in [18]). On the other hand, R116 is in close proximity to the CaM C-lobe in the channel structure, obtained in the absence of PIP2 (Figure A2A). Therefore, the observed slower activation kinetics of R116L (Figure S1) could result from effects on CaM binding to the S2–S3 linker, or from enhanced AC–AO coupling by supporting a PIP2 migration from the S2–S3 linker to the S4–S5 linker, which has been suggested to stabilize the channel open state [51]. The latter mechanism could also explain the slightly slower deactivation kinetics of R116L (Figure S1), since in KCNQ2 channels, slower deactivation might be caused by increased PIP2 binding to the S4–S5 linker, as well as by decreased PIP2 binding affinity of the S2–S3 linker [51].

In summary, we suggest that all three studied *KCNQ1* missense variants affect KCNQ1 interactions with CaM and PIP2 at different sites. This strengthens the assumption that different calcification states of CaM can affect KCNQ1 channel gating by altered PIP2 binding. With WT KCNQ1 homomers, an effect of Ca^2+^ on channel gating becomes evident with sub-physiological Ca^2+^ levels ([4,5] and this study) or with PIP2 depletion [9,12]. We can now demonstrate that the association of KCNQ1 channels with KCNE2 strongly enhances the requirement for calcified CaM for the purpose of effective channel opening. The resulting inhibition prevents high constitutive K^+^ conductance in unstimulated cells with resting Ca^2+^ levels. Our functional analysis of the three *KCNQ1* variants revealed impaired Ca^2+^ sensitivity and thereby unveiled Ca^2+^-CaM sensitivity as an important characteristic of homomeric WT KCNQ1 channels that is significantly modified by the association with KCNE2. The physiological importance of this feature of heteromeric Q1E2 channels can be inferred from the pathophysiological effects of the studied *KCNQ1* variants: the heterozygous gain-of-function variants R116L, P369L and V185M commonly lead to high-constitutive Q1E2 conductance that underlies maternally inherited gingival overgrowth, with or without postnatal growth retardation.

Study limitations. Our experiments were performed with heterologously expressed KCNQ1 channels. Hence, there might have been a relative shortage of specific molecules normally associating with native KCNQ channels in order to mediate cell type-adapted channel gating. A difference in the necessity of CaM overexpression to attain a strong activation of Q1E2 channels by high Ca^2+^ was observed between CHO cells and anterior pituitary cells. Thus, some of the described effects would probably differ in their extent between diverse native tissues, such as the stomach or thyroid gland, where KCNQ1 channels and especially heteromeric Q1E2 channels are found to be expressed [2,16,22,24,57].

## 4. Materials and Methods

### 4.1. Whole-Exome Sequencing, Variant Calling and Segregation Analysis

Genomic DNA was extracted from leukocytes using standard procedures. Whole-exome sequencing (WES) was performed on genomic DNA of individual 1, her healthy brother and healthy mother. Coding DNA fragments were enriched using the Twist Human Core Exome Plus kit (Twist Bioscience, San Francisco, CA, USA). For the other family, WES was undertaken using DNA samples from individual 3 (affected son of individual 2), individual 4 (affected sister of individual 2) and the healthy daughter of individual 2. Coding DNA fragments were enriched with the SureSelect Human All Exon V6 kit (Agilent Technologies, Santa Clara, CA, USA). Short-read sequencing was performed by CeGaT (Tübingen, Germany). Sequence reads were aligned with the human reference assembly (GRCh37/hg19) using the Burrows–Wheeler Aligner (BWA mem, v0.7.17-r1188) [58]. Genetic variants were detected with the Genome Analysis Toolkit (GATK, v3.8) [59] and annotated using ANNOVAR (v2018-04-16) [60]. Trio exome data of individual 1 and the family members were analyzed based on an autosomal dominant inheritance pattern with a heterozygous variant absent in mother and brother and not yet reported in the general population (gnomAD database v2.1.1 accessed on 6 March 2020). Additionally, trio exome data were analyzed based on an autosomal recessive inheritance model with a homozygous variant or a minimum of two heterozygous variants (minor allele frequency ≤ 0.1% and no homozygotes reported in the gnomAD database v2.1.1 accessed on 6 March 2020). The heterozygous pathogenic variant c.1106C>T/p.P369L in *KCNQ1* was identified as the disease-causing variant in individual 1.

Trio exome data of individuals 3 and 4 and the healthy daughter of individual 2 were analyzed based on an autosomal dominant inheritance pattern with a heterozygous variant that was present in individuals 3 and 4 and absent in the healthy daughter of individual 2, with a minor allele frequency of ≤0.1% in the population database (gnomAD database v2.1.1 accessed on 12 March 2020). The heterozygous variant c.553G>A/p.V185M in *KCNQ1* was identified as the disease-causing variant in individuals 3 and 4. Three out of six in silico pathogenicity prediction programs, CADD [61] (score: 24.4, pathogenic), M-CAP [62] (score: 0.165, pathogenic), MetaDome [63] (score: 0.54, slightly intolerant), Meta-SNP [64] (score: 0.467, neutral), PolyPhen2 [65] (benign), REVEL [66] (score: 0.699, pathogenic) and SIFT [67] (tolerated), predicted the variant to be pathogenic.

Sanger sequencing permitted *KCNQ1* validation and segregation analysis of c.1106C>T/p.P369L (exon 8) in individual 1 and of c.553G>A/p.V185M (exon 3) in individuals 3 and 4, as well as in individual 2 (Table A1). The *KCNQ1* variants were described according to the GenBank reference sequences NM_000218.3 and NP_000209.2.

### 4.2. Site-Directed Mutagenesis

Site-directed mutagenesis of the human KCNQ1 expression construct (GenBank reference sequence: NM_000218.3, including c.347G<T/p.R116L, c.553G>A/p.V185M and c.1106C>T/p.P369L) was performed with the QuikChange II Site-Directed Mutagenesis Kit (Agilent Technologies, Santa Clara, CA, USA) according to the manufacturer’s protocol.

### 4.3. Cell Culture

Chinese hamster ovary (CHO) cells were obtained from ATCC (American Type Culture Collection, Manassas, VA, USA). CHO cells were cultured in Dulbecco’s modified Eagle’s medium (Invitrogen GmbH, Karlsruhe, Germany), supplemented with 1% penicillin-streptomycin-glutamine (Invitrogen, Waltham, MA, USA) and 10% fetal calf serum (Biother, Kelkheim, Germany) at 37 °C in a humidified incubator (95% air, 5% CO_2_). The culture medium was changed every 2 to 3 days, and the cells were passaged when they reached confluence.

Clonal rat anterior pituitary GH_3_/B_6_ cells [25,39] were kindly provided by Dr. A. Tixier-Vidal, Collège de France, Paris, France. GH_3_/B_6_ cells were cultured in Ham’s F10 medium (Sigma-Aldrich, St. Louis, MO, USA), supplemented with 15% horse serum (Gibco/Invitrogen, Waltham, MA, USA), 2.5% fetal calf serum (Biother, Kelkheim, Germany) and 0.5% L-glutamine (Sigma-Aldrich, St. Louis, MO, USA). The culture medium was changed every 2–3 days. The cells were grown at 37 °C in an atmosphere of 95% air and 5% CO_2_ and passaged every 5–7 days.

### 4.4. Heterologous Expression

The following plasmids were used for heterologous expression: mutant (R116L, V185M and P369L) and WT human KCNQ1 in pcDNA3.1; rat KCNE1 (GenBank reference sequence: NM_012973), KCNE2 (GenBank reference sequence: AF071003) and KCNE3 (GenBank reference sequence: AJ271742) in pcDNA3; EGFP-N1 (Clontech/Takara Bio, Heidelberg, Germany) in pcDNA3; and human EYFP-linked calmodulin (EYFP-hCaM, a kind gift from Emanuel Strehler [68], Addgene plasmid #47603) in pEYFP.

*Transfection.* The CHO cells or GH_3_/B_6_ cells, once plated on plastic coverslips in 35 mm culture dishes, were transfected with the relevant cDNAs using LipofectAMINE 2000 reagent (Invitrogen, Waltham, MA, USA), according to the manufacturer’s instructions. For the expression of WT and mutant KCNQ1 channels, channel cDNA (final concentration: 1 µg/mL) was applied, together with cDNA encoding EGFP-N1 (0.4 µg/mL) or EYFP-hCaM (1 µg/mL). In part of the experiments, KCNE2 was co-transfected (2.5 µg/mL). Patch clamp recordings were performed two days after transfection.

*Microinjection.* The microinjection technique was used for the co-expression of WT or V185M KCNQ1 channels with either KCNE1 or KCNE3 to encourage saturation of KCNQ1 channels with the KCNE beta subunits. The CHO cells were plated on glass coverslips coated with poly-D-lysine (Sigma-Aldrich, St. Louis, MO, USA) in 35 mm plastic culture dishes (Nunc/Thermo Fisher Scientific, Wiesbaden, Germany) and microinjected using an Eppendorf Transjector 5246 (Eppendorf, Hamburg, Germany) with cDNA encoding WT or mutant KCNQ1 (50 ng/µL), together with cDNA encoding KCNE1 or KCNE3 (250 ng/µL). To enable the detection of successfully expressing CHO cells, EGFP-N1 (20 ng/µL) was always co-expressed. Electrophysiological experiments were performed 1 day after cell injection.

### 4.5. Solutions and Special Experimental Conditions

The standard external Ringer solution (ECS: extracellular solution) contained (in mM): NaCl 140, KCl 5, MgCl_2_ 0.8, CaCl_2_ 1, HEPES 10 and glucose 5, with the pH adjusted to 7.35 with NaOH. The “low Ca^2+^” EGTA-Ringer solution contained (in mM): NaCl 140, KCl 5, MgCl_2_ 0.8, CaCl_2_ 1, EGTA 2.5, HEPES 10 and glucose 5, with the pH adjusted to 7.35 with NaOH. The standard “low Ca^2+^” pipette solution (ICS: intracellular solution) contained (in mM): K-aspartate 125, KCl 20, MgCl_2_ 1, CaCl_2_ 1, HEPES 10, EGTA 10 and K_2_-ATP 5, with the pH adjusted to 7.2 with KOH (Maxchelator: about 20 nM free Ca^2+^ at 21 °C). The “high Ca^2+^” ICS contained (in mM): K-aspartate 130, MgCl_2_ 1, CaCl_2_ 9.76, HEPES 10, EGTA 10 and K_2_-ATP 5, with the pH adjusted to 7.2 with KOH (Maxchelator: 5 µM free Ca^2+^ at 21 °C). The following Maxchelator version was used for calculation: https://somapp.ucdmc.ucdavis.edu/pharmacology/bers/maxchelator/CaMgATPEGTA-NIST.htm (accessed on 28 July 2020).

“PIP2” experiments: PI(4,5)P2 DiC8 was purchased from Echelon Biosciences Inc. (Salt Lake City, UT, USA) and the aliquots of the reconstituted PI(4,5)P2 DiC8 (2 mM in a. dest.) were stored at −80 °C and used within 2 days. Additionally, 100 µM-PIP2-containing pipette solutions were freshly prepared and sonicated just before an experiment, using standard intracellular solution.

“BAPTA” experiments: Two hours prior to the start of the recording, the membrane-permeable form of BAPTA, BAPTA-AM (1,2-Bis(2-aminophenoxy)ethane-N,N,N′,N′-tetra-acetic acid tetrakis(acetoxymethyl ester); Sigma-Aldrich, St. Louis, MO, USA), was added to the cell culture medium to reach a final concentration of 10 µM (using stock solutions of 10 mM BAPTA-AM in DMSO), and the cells were incubated for 1 h at 37 °C. To remove unmetabolized BAPTA-AM, which might possibly exert unspecific effects on KCNQ1 channels [69,70], the incubation period was followed by a 1-h wash period in normal CHO culture medium in the incubator at 37 °C.

### 4.6. Electrophysiology

Membrane currents of KCNQ1 channel-expressing CHO cells or GH_3_/B_6_ cells were recorded in the conventional whole-cell configuration of the patch-clamp technique. Patch pipettes were made from 1.5 mm-diameter borosilicate glass capillaries with resistances of 3.5 to 4 MΩ when filled with standard (low chloride) solution. Data were low-pass filtered at 3 kHz and compensated for both fast and slow capacity transients prior to the pulse protocols. The series resistance compensation was as high as possible (60 to 90%). All data were online-corrected for a liquid junction potential of about −13 mV for an aspartate-based intracellular solution. An EPC-9 patch clamp amplifier was used in combination with the PATCHMASTER stimulation and data acquisition software (HEKA Elektronik, Lamprecht, Germany). Electrophysiological recordings were performed at room temperature.

### 4.7. Data Analysis

Patch-clamp data processing was performed with FITMASTER (HEKA Elektronik, Lamprecht, Germany), Excel (Microsoft Corp., Seattle, Washington) and SigmaPlot 11.0 (SPSS Inc., Chicago, IL, USA). G–V relation: To assess the voltage dependence of isochronal KCNQ1 channel activation from current recordings using a double pulse protocol, the normalized data of instantaneous P2 current amplitudes were fitted with a Boltzmann equation: y = c + d/(1 + exp (−(*V* − *V*_0_._5_)/*k*)), where *V*_0_._5_ is the potential of half-maximal voltage-dependent KCNQ1 channel activation and *k* is the slope factor. The WT KCNQ1 data obtained after more than five minutes in the whole-cell configuration were fitted with the sum of two Boltzmann terms, also yielding *V*_0_._5_ and *k* values of an additional high-voltage-activated (HVA) current component. The time course of current activation and the time course of KCNQ1 current decay due to deactivation were fitted with double exponential functions, yielding fast (τ_fast_) and slow time constants (τ_slow_) of activation or deactivation, as well as the amplitudes of the fast and slow current components.

### 4.8. Statistics

Most experimental data are presented as means ± SEM, with n representing the number of experiments using different cells. In these experiments, a statistical comparison of two groups was performed with Student’s two-tailed t-test. To compare more than one group with the control WT KCNQ1 data, one-way ANOVA with post hoc Bonferroni t-test was used to test for significant differences. Current amplitudes and, accordingly, current densities often showed high variability, and such data are presented as box plot (Cleveland method: boxes indicate the 25% to 75% range, whiskers indicate the 10/90 percentiles and points normally indicate the 5/95 percentiles; if points indicate all outliers, this is explicitly mentioned in the figure legend; the median is always shown as a line). Statistical analyses of the data sets that failed to pass the Shapiro–Wilk normality test were performed using nonparametric tests. Statistical analyses of the two groups were performed using the Mann–Whitney rank sum test. Statistical analyses of more than two groups were performed with the nonparametric ANOVA for the ranks (Kruskal–Wallis test) with post hoc Dunn’s test to identify the significantly differing groups. In all statistical analyses, α was set to 0.05, and statistical significance was indicated by * *p* < 0.05, ** *p* < 0.01 and *** *p* < 0.001. The statistical testing was performed with SigmaPlot 11.0 and SigmaPlot 13.0 (SPSS Inc., Chicago, Illinois).

### 4.9. Model Generation

The extended Markov Model, considering the Ca^2+^ sensitivity of WT KCNQ1 channels (Figure A1 in Appendix B), was established with the free software program MarkovEditor, provided by Michael Pusch [71]. This model is an extension of a five-state kinetic KCNQ1 model [34] with parameters adapted to KCNQ1 currents recorded in CHO cells.

The structure models of the KCNQ1-CaM complex in the PIP2-free and the PIP2-bound states (Figure A2 in Appendix B) are based on pdb 6V00 and pdb 6V01 [14] and were generated using the program UCSF Chimera 1.14 (San Francisco, CA, USA).

## 5. Conclusions

With the identification of a recurrent (P369L) and a novel (V185M) *KCNQ1* variant (Table A1) and our functional analyses, we confirm that specific heterozygous *KCNQ1* missense variants cause KCNQ1-KCNE2 (Q1E2) channel gain-of-function and underlie a particular disorder characterized by gingival overgrowth with or without postnatal growth retardation [20]. Our data provide new insights into the Ca^2+^ sensitivity of wild-type KCNQ1 channels. We showed that KCNE2, when associated with KCNQ1, exerts a drastic sustained inhibition of the Q1E2 channel availability, and that KCNQ1-bound CaM requires high Ca^2+^ to effectively relieve this inhibition. In contrast, the three analyzed KCNQ1 mutants (R116L, V185M and P369L) exhibit impaired sensitivity to changes in intracellular Ca^2+^, as homomeric channels, and mediate a significantly increased resting K^+^ conductance when associated with KCNE2. The impaired Ca^2+^ sensitivity of the mutant channels causes the prominent gain-of-function of the Q1E2 channel complex that likely underlies the specific clinical features of the affected individuals, including gingival overgrowth. Remarkably, gain-of-function variants of *KCNH1*, *KCNK4* and *KCNN3* encoding other K^+^ channels also cause gingival enlargement in addition to other clinical features. We have previously suggested combining these phenotypes into a subgroup of potassium channelopathies [30], as all functionally studied variants lead to increased K^+^ conductance of the mutant channels [26,27,28,29]. Here, we propose to add *KCNQ1* to this group of K^+^ channelopathies. Future studies are required to resolve the question of whether the different K^+^ channel variants affect the same cell type(s) to induce gingival hyperplasia. A common pathomechanism could help to develop an adequate drug therapy.

## Data Availability

Whole-exome sequencing data are not publicly available due to privacy or ethical restrictions. All other study data are included in the article, Appendix A and Appendix B and the Supplementary Materials file.

## Supplementary Materials

The following supporting information can be downloaded at: https://www.mdpi.com/article/10.3390/ijms23179690/s1.

## Appendix A

**Table A1:** Clinical features in individuals with a heterozygous *KCNQ1* pathogenic variant identified in this study.

Individual	1	2	3 (Son of Individual 2)	4 (Sister of Individual 2)
Gene	*KCNQ1*	*KCNQ1*	*KCNQ1*	*KCNQ1*
mRNA reference number	NM_000218.3	NM_000218.3	NM_000218.3	NM_000218.3
Variant	c.1106C>T p.Pro369Leu	c.553G>A p.Val185Met	c.553G>A p.Val185Met	c.553G>A p.Val185Met
Worldwide MAF of the variant (gnomAD 2.1.1)	Absent	0.00001	0.00001	0.00001
Origin	Unknown	unknown	Maternally inherited	unknown
Nationality	Caucasian (Egyptian)	Caucasian (Portuguese)	Caucasian (Portuguese)	Caucasian (Portuguese)
Sex	Female	Female	Male	Female
Age at last examination	5 years and 9 months	38 years	16 years and 11 months	38 years
Birth weight	3500 g (full term) (0.57 z)	ND	ND	ND
Birth length	48 cm (−0.6 z)	ND	ND	ND
OFC at birth	ND	ND	ND	ND
Weight at last examination	16 kg (−1.91 z)	68.2 kg BMI: 26.2	55 kg (−1.53 z)	90 kg BMI: 35.2
Height at last examination	102 cm (−2.82 z)	161 cm (−1.1 z)	165 cm (−2 z)	160 cm (−1.26 z)
OFC at last examination	51 cm (0.05 z)	54 cm (−1.03 z)	55.5 cm (−0.77 z)	ND
Craniofacial dysmorphism	Coarse face, upslanted palpebral fissures, bulbous nose, thickened ears with fleshy lobes, wide open mouth, high-arched and narrow palate, failure of eruption of primary teeth	Long face, broad nasal tip, full lips, retrognathia	Long face, broad nasal tip, full lips	Coarse face, thick eyebrows, retrognathia, high-arched palate
Gingival overgrowth	+ (progressive since early infancy)	+ (since childhood)	+ (since birth)	+ (since infancy)
Pituitary hormone deficiencies	T3, T4, and thyroid-stimulating hormone normal, IGF-1 very low, GH deficiency (maximum GH after clonidine and L dopa: 0.76 ng/mL; normal response >10)	ND	ND	ND
Developmental delay	−	−	+	Learning difficulties
MRI scan	Cavum septum pellucidum and cavum vergae, a small simple choroid plexus cyst seen at the trigone of the right lateral ventricle, otherwise normal	ND	ND	ND
Cardiovascularfeatures	Normal echocardiogram and electrocardiogram	Normal echocardio-gram and electrocardio-gram	ND	Normal electrocardiogram
Other anomalies	Long fingers, hypertrichosis on back, abnormality of the voice	−	Long fingers	Brachydactyly

+, feature present; −, feature absent; BMI, body mass index; GH, growth hormone; MAF, minor allele frequency; MRI, magnetic resonance imaging; ND, no data; z, z-score.

## Appendix B

Appendix B contains two figures related to the Discussion.
