# Supplementary material for: Clinically Relevant KCNQ1 Variants Causing KCNQ1-KCNE2 Gain-of-Function Affect the Ca2+ Sensitivity of the Channel
Source: Int J Mol Sci. 2022 Aug 26;23(17):9690. doi: 10.3390/ijms23179690 (PMC9456291; doi:10.3390/ijms23179690)
Supplement: Supplementary file 1 [file ijms-23-09690-s001.zip › ijms-1863899-supplementary.pdf]

Supporting Information for

Clinically Relevant *KCNQ1* Variants Causing KCNQ1-KCNE2 Gain-of-Function Affect the Ca^{2+} Sensitivity of the Channel

Christiane K. Bauer [1]*, Tess Holling [2], Denise Horn [3], Mário Nôro Laço [4], Ebtesam Abdalla [5,6], Omneya Magdy Omar [7], Malik Alawi [8] and Kerstin Kutsche [2]

[1] Department of Cellular and Integrative Physiology, University Medical Center Hamburg-Eppendorf, 20246 Hamburg, Germany
[2] Institute of Human Genetics, University Medical Center Hamburg-Eppendorf, 20246 Hamburg, Germany; t.holling@uke.de (T.H.); k.kutsche@uke.de (K.K.)
[3] Department of Medical Genetics and Human Genetics, Charité - Universitätsmedizin Berlin, corporate member of Freie Universität Berlin, Humboldt Universität zu Berlin, and Berlin Institute of Health, 13353 Berlin, Germany; denise.horn@charite.de
[4] Medical Genetics Unit, Hospital Pediátrico, Centro Hospitalar e Universitário de Coimbra, 3004-561 Coimbra, Portugal; noro.laco@gmail.com
[5] Department of Human Genetics, Medical Research Institute, Alexandria University, 5422031 Alexandria, Egypt; drebtesamabdalla@yahoo.com
[6] Genetics Department, Armed Forces College of Medicine (AFCM), 4460015 Cairo, Egypt
[7] Department of Pediatrics, Faculty of Medicine, Alexandria University, 5422031 Alexandria, Egypt; o_magdy09@alexmed.edu.eg
[8] Bioinformatics Core, University Medical Center Hamburg-Eppendorf, 20246 Hamburg, Germany; m.alawi@uke.de
* Correspondence: c.bauer@uke.de

This file includes Figures S1-S6

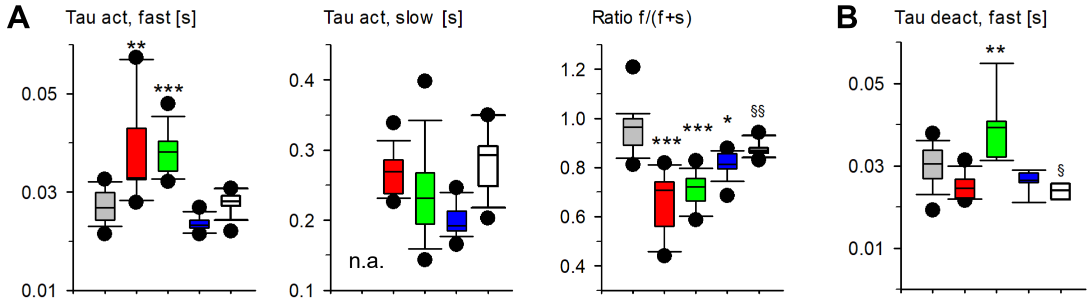

Act +40 mV	τ_fast (ms)	τ_slow (ms)	Ratio f/(f+s)	n		Deact -120mV	τ_fast (ms)	Ratio f/(f+s)	n
WT KCNQ1	27.0 ± 0.7	n.a.	0.96 ± 0.02	24	(grey)	WT KCNQ1	30.0 ± 1.5	0.96 ± 0.01	12
V185M	38.0 ± 3.4	268 ± 10	0.66 ± 0.04	10	(red)	V185M	25.1 ± 1.0	0.97 ± 0.01	10
R116L	38.2 ± 1.4	241 ± 21	0.71 ± 0.02	12	(green)	R116L	39.9 ± 3.7	0.97 ± 0.02	6
P369L	23.6 ± 0.5	201 ± 7	0.81 ± 0.02	11	(blue)	P369L	26.2 ± 1.2	0.96 ± 0.004	6
WT+CaM+Ca	27.9 ± 1.2	284 ± 14	0.87 ± 0.01	11	(white)	WT+CaM+Ca	23.8 ± 1.2	0.91 ± 0.03	4

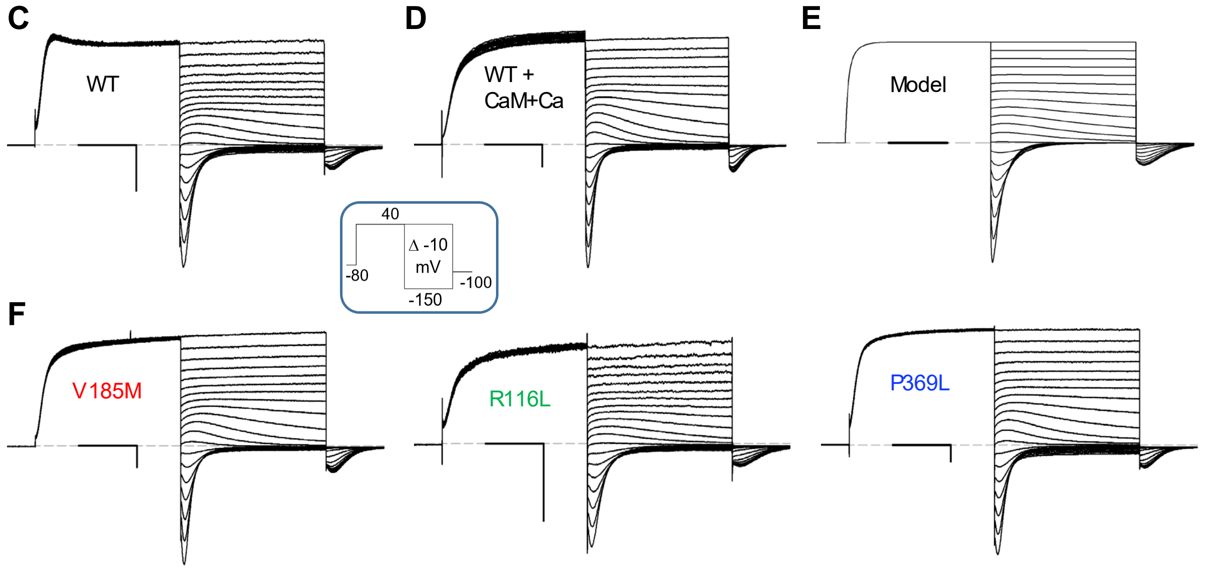

Figure S1. Activation and deactivation kinetics of WT and mutant KCNQ1 channels.

(A) The time course of KCNQ1 current activation upon a depolarization to +40 mV from a holding potential of −80 mV was analyzed by fitting a double exponential function to the recorded current traces short after establishment of the whole-cell configuration. Due to the variable form of the WT KCNQ1 currents recorded with standard ICS and ECS including even slight fast inactivation (ratio of the fast activating current component >1), values of Tau_slow are not given (n.a., not applicable). **(B)** The time course of KCNQ1 current decay upon a hyperpolarizing pulse to −120 mV following a 500 ms depolarization to +40 mV was analyzed by fitting a double exponential function to the recorded current traces after the initial "hook". Due to the very small amplitude of the slowly decaying current component, only values for Tau_fast are shown. Number of experiments and the color code used in the box plots are given in the table with means ± SEM. Asterisks indicate significant differences between WT and mutant KCNQ1 channel data: ***$p < 0.001$; **$p < 0.01$; *$p < 0.05$. For WT KCNQ1 channels, data from experiments with co-expressed CaM and high-Ca^{2+} ICS are also included. Significant differences between these two WT data sets are indicated by §§ $p < 0.01$ and § $p < 0.05$. **(C)** Overlay of WT KCNQ1 current traces recorded with the indicated deactivation protocol using standard solutions (ECS: standard Ringer, ICS: low Ca^{2+}). **(D)** WT KCNQ1 currents recorded from a cell co-expressing CaM and using the high-Ca^{2+} ICS. **(E)** Simulated WT KCNQ1 currents generated by the extended Markov model using low-Ca^{2+} parameter (see Figure A1 in Appendix B). **(F)** Current traces of the mutant KCNQ1 channels recorded with standard ECS and ICS solutions. Scale bars in (C-F) denote 0.2 s and 0.5 nA.

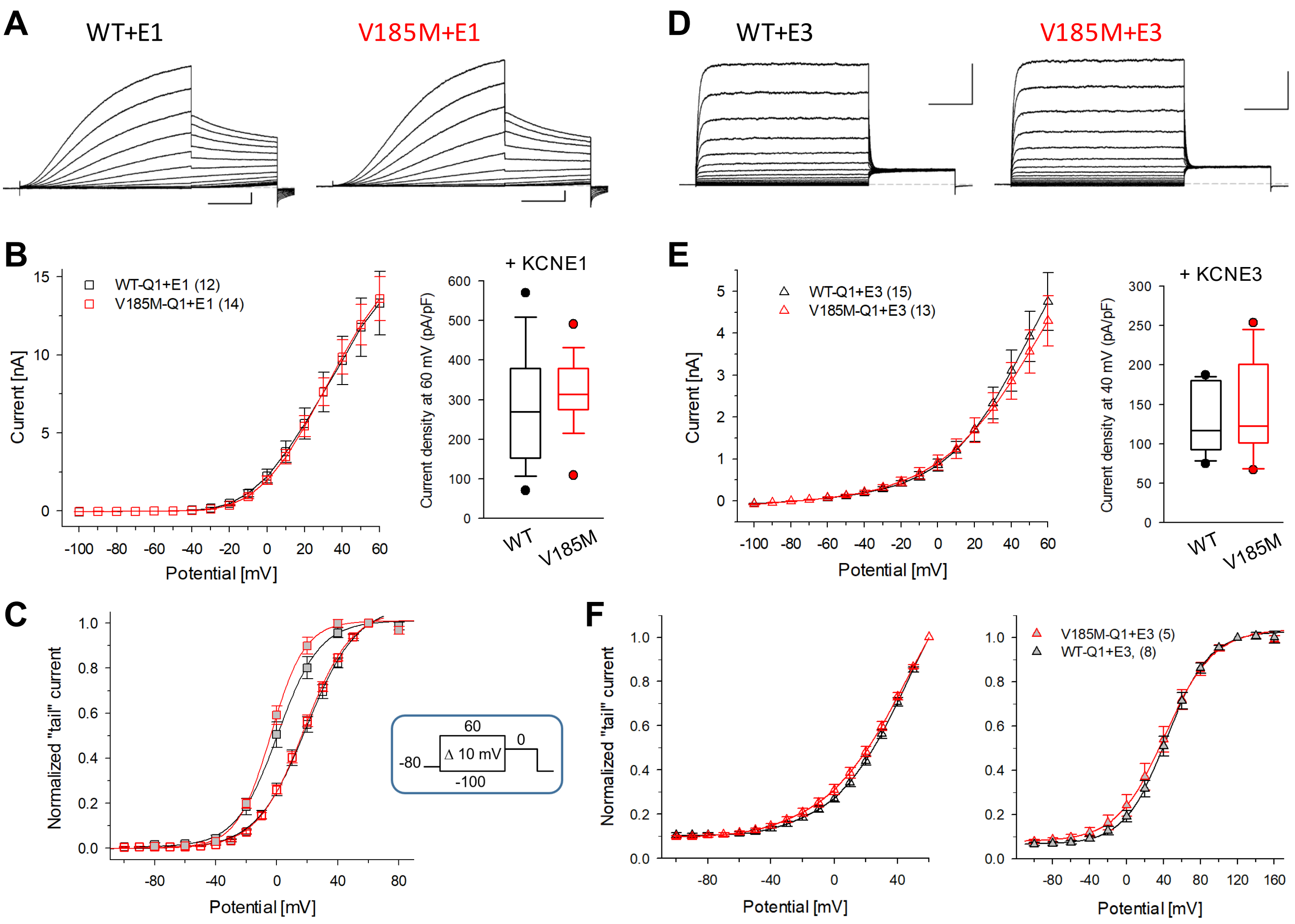

Experimental condition	n	V0.5 (mV)	SEM	p *versus* WT	k (mV)	SEM	p *versus* WT
WT-Q1 + E1 (2s)	12	19.75	2.67		15.79	0.38	
WT-Q1 + E1 (6s)	11	1.88	3.29		13.00	0.58	
V185M-Q1 + E1 (2s)	14	18.19	1.85	0.626	15.02	0.32	0.131
V185M-Q1 + E1 (6s)	11	-2.56	1.75	0.248	11.63	0.55	0.100
WT-Q1 + E3	8	43.74	4.26		22.27	0.71	
V185M-Q1 + E3	5	41.22	6.53	0.740	25.71	1.54	0.042

Figure S2. No striking effect of V185M in Q1E1 and Q1E3 heteromers.

Upon co-expression of KCNQ1 with KCNE1 (**A-C**) or KCNE3 (**D-F**), no significant effects of the V185M mutation were detected on voltage-dependent activation or amplitude of the recorded currents. **(A,D)** typical currents recorded with the standard activation protocol shown at the bottom. Scale bars denote 0.5 s and 1 nA. **(B,E)** IV relation and distribution of current densities. **(C,F)** GV relation measured as normalized instantaneous P2 current amplitude. To yield saturating values, additional data obtained with 6 s variable P1 pulses are included for Q1E1 (**C**; squares with grey filling) and an extended voltage range of 2 s P1 pulses up to 160 mV was used for Q1E3 (**F**, right panel). Numbers of experiments performed with the standard activation protocol (see inset) are given in (B) and (E). Channel activation parameter are summarized in the table.

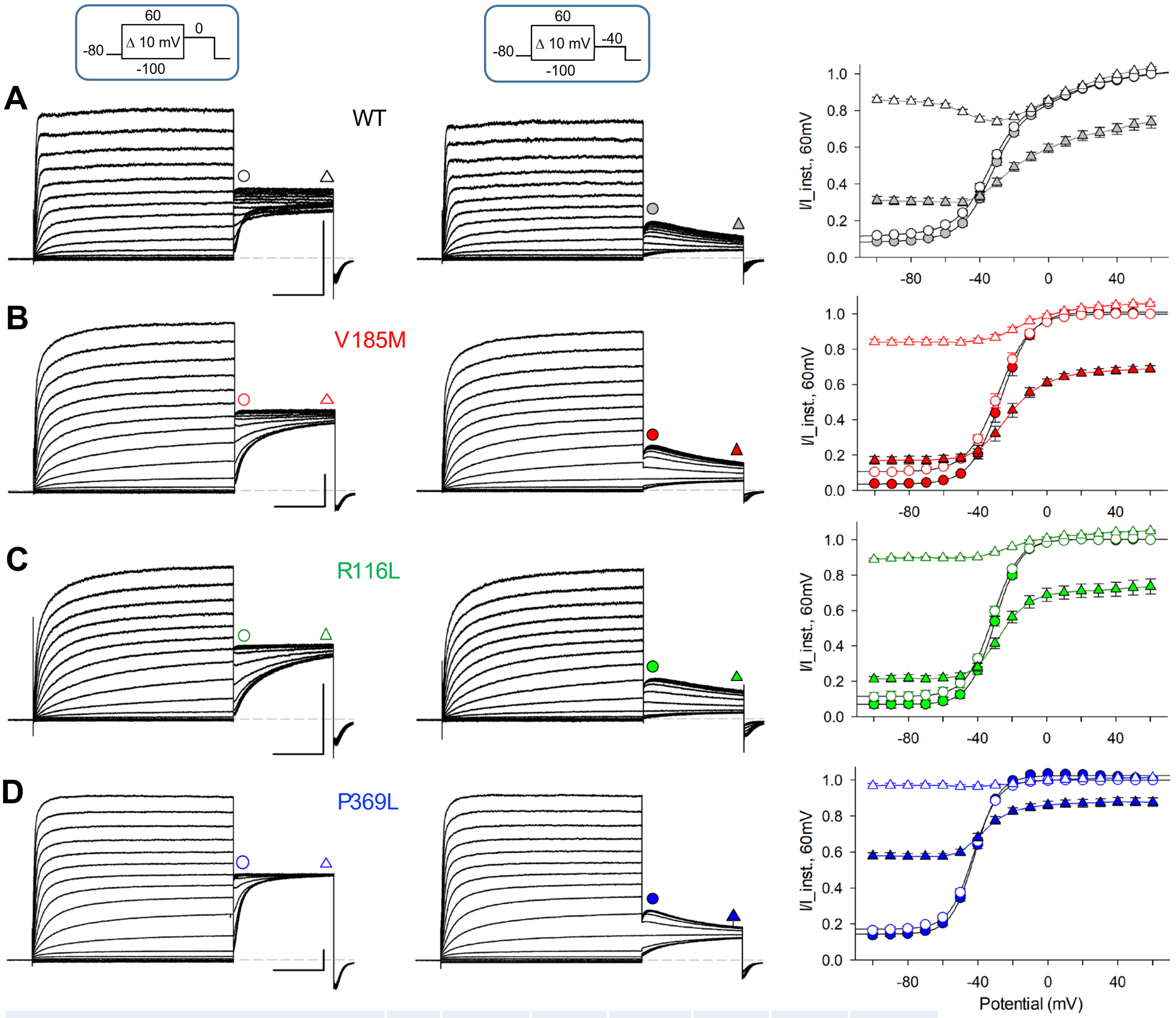

Experimental condition	n	$V_{0.5}$ (mV)	SEM	p value	k (mV)	SEM	p value
WT (> 5min) P2 0 mV; component 1	13	-34.96	1.09		8.52	0.28	
double Boltzmann fit: component 2 (HVA)		2.83	2.88		25.86	2.16	
WT (>5min) P2 -40 mV; component 1		-34.17	0.99	**0.050**	7.62	0.33	0.112
double Boltzmann fit: component 2 (HVA)		1.41	3.37	0.793	24.88	3.88	0.812
V185M (> 5min) P2 0 mV	6	-27.60	1.97		8.72	0.23	
V185M (> 5min) P2 -40 mV		-26.98	1.92	0.236	8.37	0.28	**0.042**
R116L (> 5min) P2 0 mV	6	-31.33	0.60		7.65	0.12	
R116L (> 5min) P2 -40 mV		-29.94	0.67	**0.027**	7.43	0.21	0.209
P369L (> 5min) P2 0 mV	10	-42.51	0.56		6.79	0.11	
P369L (> 5min) P2 -40 mV		-41.78	0.64	**0.016**	6.69	0.08	0.207

p: *versus* respective values obtained with P2 pulse of 0 mV (*t*-test; paired data)

Figure S3. P2 potentials of either 0 mV or −40 mV yield only small differences in the voltage dependence of KCNQ1 activation.

Left and middle panels: Typical families of current traces after more than 5 min in whole-cell configuration for WT **(A)** and the indicated mutant KCNQ1 channels **(B-D)**; scale bars indicate 0.5 s and 0.5 nA. **(A-D)** right panels: Mean (±SEM) normalized instantaneous (circles: GV data) and late (triangles) P2 current amplitudes as function of P1 potential for P2 = 0 mV (open symbols) and P2 = −40 mV (filled symbols). The ″cross-over phenomenon″ of WT channels is not obvious with −40 mV P2 pulses. Standard ICS (low Ca^{2+}) and ECS. Channel activation parameter and numbers of experiments are summarized in the table.

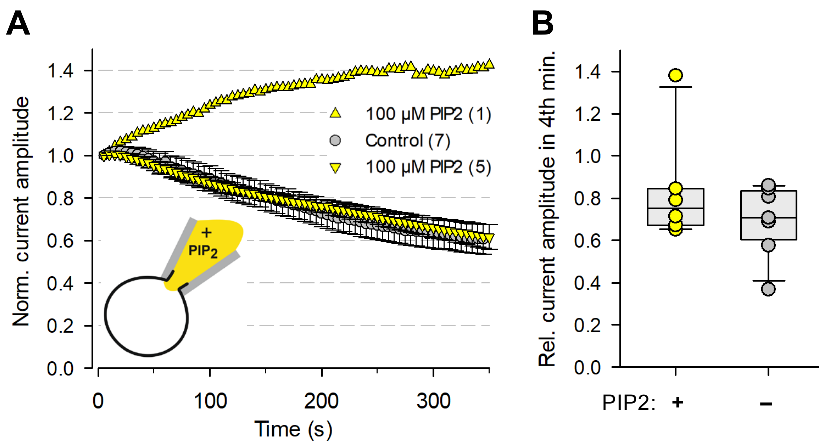

Figure S4. Washout of PIP2 is not the major cause of early KCNQ1 current run-down.

(A) Addition of PIP2 to the pipette solution did not prevent current run-down in 5 of 6 cells expressing WT KCNQ1 channels. Current increase occurred in 1 of 6 experiments (△), whereas in the other five experiments, the amount of current run-down did not differ from that observed in parallel control experiments (n = 7). Time course of mean (± SEM) WT-KCNQ1 current amplitudes traced every 5 s with a 500 ms test pulse to +40 mV. Current amplitudes were normalized to the initial value. **(B)** The boxplot shows relative current amplitudes 3.5 to 4 min after the first measurement in the whole-cell configuration; points indicate all individual values. ICS: standard 20 nM Ca^{2+} and 5 mM ATP with or without addition of 100 µM PIP2DiC8.

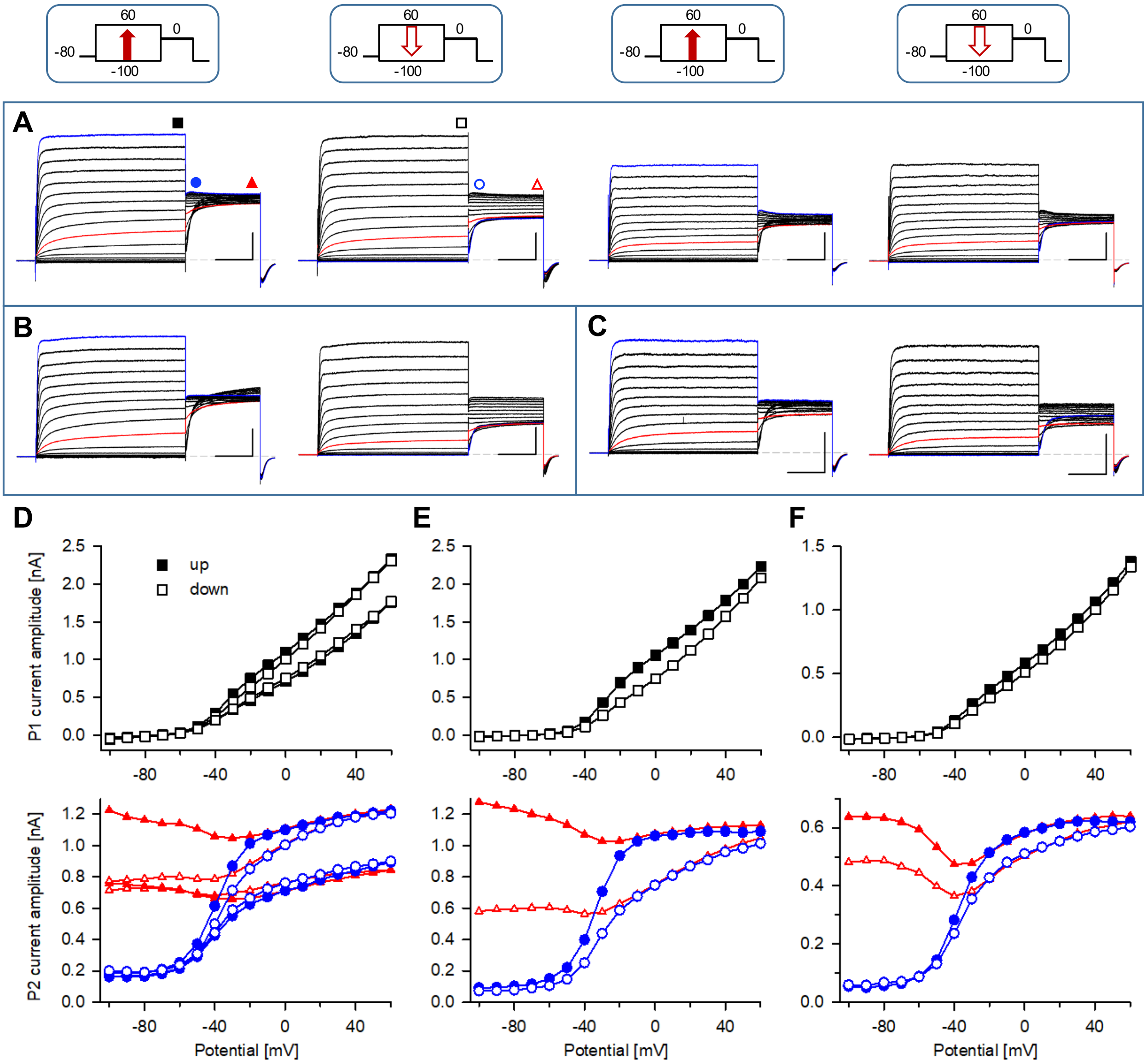

Figure S5. Run-down during ascending or descending test pulse protocols oppositely affects the detectability of a high-voltage-activated (HVA) current component and the cross-over phenomenon in WT KCNQ1.

(A-C) Families of WT KCNQ1 current traces from three different cells (a, b, c, respectively) consecutively recorded with the standard activation protocol and the inverted protocol with descending test pulses. The trace recorded at last is shown in blue, the trace obtained with the P1 pulse to −30 mV is shown in red. Scale bars denote 0.5 s and 0.5 nA. **(D-F)** IV-plots (top panels) and initial (blue "GV" data) as well as late (red triangles) P2 current amplitudes (bottom panels) determined for cell a (**D**), cell b (**E**) and cell c (**F**); meaning of symbols is shown in (**A**). Run-down is well indicated by a divergence of the red late P2 curves. Cell "a" (**A** and **D**): typical early run-down, followed by a period with relatively stable current amplitudes. Cell "b" (**B** and **E**): pronounced run-down during the initial standard activation protocol obscured the HVA current component and overemphasized "cross-over"; further run-down during the inverted protocol overemphasized the HVA current component and obscured the "cross-over" phenomenon. Recordings from cell "c" (**C** and **F**) with moderate run-down point to a small HVA current component and a pronounced cross-over phenomenon.

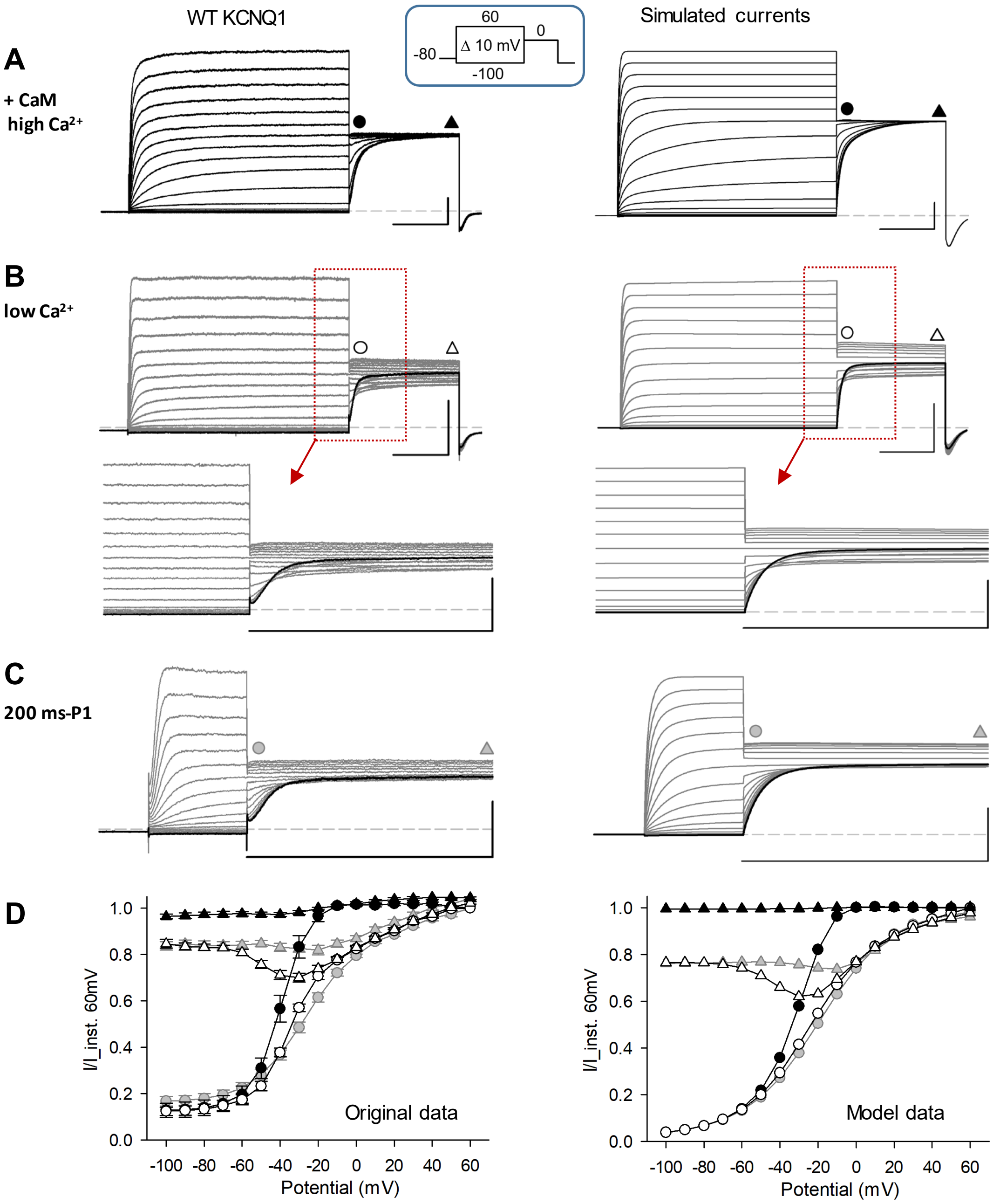

Figure S6. Model simulations reproduce essential phenomena of the Ca-CaM sensitivity of the KCNQ1 WT channel.

Comparison of original KCNQ1 data (left panels) and data derived from a Markov model (right panels; Markov model: see Figure A1 in Appendix B). **(A)** Co-expression of WT KCNQ1 with CaM and use of ICS with 5 µM Ca^{2+}: no cross-over of current traces and single component GV (D). **(B)** WT KCNQ1 and endogenous CaM: current traces recorded with standard low-Ca^{2+} ICS exhibit pronounced cross-over during the P2 pulse and GV curves show an additional high-voltage-activated component (D). In (B) and (C), the trace obtained with the P1 pulse to −100 mV is shown in black. **(C)** Shortening the variable P1 pulse from standard 2 s to 200 ms mostly eliminated the cross-over of current traces, but not the high-voltage-activated component. Scale bars in (A-C) correspond to 0.5 s and 0.5 nA (or 1 arbitrary unit). **(D)** IV-Plots of instantaneous (circles) and late (triangles) normalized P2 current amplitudes as function of the P1 potential. Meaning of symbols is indicated in (A-C). Left panel: means ± SEM with $n = 7$ in each of the three data sets; KCNQ1 WT data obtained with 2 s and 200 ms P1 pulses were recorded from the same cells using standard ICS (see example in B and C).
